# Highly selective oxidation of benzene to phenol with air at room temperature promoted by water

**DOI:** 10.1038/s41467-023-40160-w

**Published:** 2023-07-22

**Authors:** Jijia Xie, Xiyi Li, Jian Guo, Lei Luo, Juan J. Delgado, Natalia Martsinovich, Junwang Tang

**Affiliations:** 1 Department of Chemical Engineering, University College London, London, WC1E 7JE UK; 2 Department of Chemistry, University College London, London, WC1H 0AJ UK; 3 Key Lab of Synthetic and Natural Functional, Molecule Chemistry of Ministry of Education, the Energy and Catalysis Hub, College of Chemistry and Materials Science, Northwest University, Xi’an, 710127 China; 4 Departamento de Ciencia de los Materiales e Ingeniería Metalúrgica y Química Inorgánica, Facultad de Ciencias, Universidad de Cádiz, Campus Rio San Pedro, 11510 Puerto Real, Cádiz Spain; 5 IMEYMAT, Instituto de Microscopía Electrónica y Materiales, Puerto Real, 11510 Spain; 6 Department of Chemistry, University of Sheffield, Sheffield, S3 7HF UK; 7 Industrial Catalysis Center, Department of Chemical Engineering, Tsinghua University, Beijing, 100084 China; 8 Present Address: Sinopec Beijing Research Institute of Chemical Industry, Sinopec Group, Beijing, 100013 China; 9 Present Address: College of Physics, Sichuan University, Chengdu, Sichuan 610064 China; 10 Present Address: State Key Laboratory of Catalysis, Dalian Institute of Chemical Physics, The Collaborative Innovation Centre of Chemistry for Energy Materials (iChEM), Dalian National Laboratory for Clean Energy, Chinese Academy of Sciences, Dalian, Liaoning 116023 China

**Keywords:** Photocatalysis, Chemical engineering

## Abstract

Phenol is one of the most important fine chemical intermediates in the synthesis of plastics and drugs with a market size of *ca*. $30b^1^ and the commercial production is via a two-step selective oxidation of benzene, requiring high energy input (high temperature and high pressure) in the presence of a corrosive acidic medium, and causing serious environmental issues^2–5^. Here we present a four-phase interface strategy with well-designed Pd@Cu nanoarchitecture decorated TiO_2_ as a catalyst in a suspension system. The optimised catalyst leads to a turnover number of 16,000–100,000 for phenol generation with respect to the active sites and an excellent selectivity of *ca*. 93%. Such unprecedented results are attributed to the efficient activation of benzene by the atomically Cu coated Pd nanoarchitecture, enhanced charge separation, and an oxidant-lean environment. The rational design of catalyst and reaction system provides a green pathway for the selective conversion of symmetric organic molecules.

## Abstract (web summary)

Phenol is one of the most important fine chemicals, but its synthesis is highly energy intensive. Here, the authors report an economical pathway for the selective photocatalytic conversion of benzene to phenol in high yield and selectivity by Pd@Cu/TiO_2_ catalyst using with air as oxidant.

## Introduction

Direct oxidation of highly symmetric benzene to phenol by the most abundant oxidant O_2_ gas with high efficiency or turnover number (TON) is highly challenging due to several critical issues: (i) the benzene molecules are extremely stable owing to the conjugated π bond and highly symmetric structure, (ii) O_2_ gas as a low-cost oxidant also requires high activation energy to generate active radicals and (iii) the expected product phenol is much more active than benzene, favouring the over-oxidation products (e.g. polyphenols, benzoquinone, CO_2_, etc.)^2–5^. Substantial effort has been put into the development of a thermal catalyst for direct benzene hydroxylation. For instance, the Pd membrane was reported to convert benzene to phenol using H_2_ and O_2_ as reactants operated at 150 °C, resulting in a TON of 1500 after a 10-h reaction^2^. Fe-ZSM and Cs-β zeolite were reported to activate the C–H bond of benzene and produced phenol using N_2_O as an oxidant at 400 °C^6,7^. Recently, it was reported that Fe-N_x_C_y_^8^ and FeOCl^9^ made it possible to lower the reaction temperature to 30–60 °C with a phenol yield of 2.68 mmol/g/h when using H_2_O_2_ as the oxidant. One can see that the key in a thermal catalytic process is to use a weaker oxidant e.g. NO_2_^6,7^ or H_2_O_2_ (or a mixture of H_2_ and O_2_)^8–12^ to avoid over-oxidation, but such oxidants are expensive and corrosive in addition to their detrimental impact on the environment.

It was also found that in most cases acetonitrile had to be used as the solvent to achieve a uniform mixture of benzene and the catalysts. This solvent is volatile, toxic and flammable compared to green and abundant water. However, using water is not beneficial for benzene conversion due to the very low solubility of benzene in water. Furthermore, using a single organic solvent (e.g. acetonitrile) or a single gas phase can maximise the contact between reactants and catalysts, while also leading to mixing of over-supplied oxidants and unstable phenol products. From the thermodynamic viewpoint, the expected unstable product would be readily over-oxidised in this well-mixed phase under the harsh reaction conditions^2–5^. To enable the one-step oxidation of benzene and further improve the selectivity of the expected phenol product, a local environment should be modulated to avoid the oxidant-rich environment. On the other hand, photocatalysis can operate under ambient conditions and it has been proved to be a promising strategy in the activation of stable molecules, e.g. H_2_O^13,14^, CO_2_^15,16^, and N_2_^17^. This characteristic of photocatalysis would moderate the harsh reaction conditions required, thus avoiding a shift from the exothermic reaction of benzene oxidation forming the oxidation products to the reverse reaction of forming the reactants^18^. We recently also demonstrated that photocatalysis could achieve highly selective activation of another symmetric molecule, CH_4_, by peroxide species in water solution^19^ or even ambient air^20^. Decorated CdWO_4_ micro-rods were also reported to activate benzene to phenol at room temperature although the synthesis rate was around two orders of magnitude lower than that achieved by an efficient thermal catalyst (*ca*. 0.19 mmol/g/h)^21^. Therefore, a photocatalytic approach is potentially feasible for efficient and selective benzene hydroxylation to phenol in one step using water as a solvent if an efficient photocatalyst could be discovered, which to the best of our knowledge has not been reported so far.

As a highly stable symmetric molecule, the activation of benzene requires a high oxidation potential of *ca*. 2.5 eV^22^. Therefore, the candidate photocatalysts should have a valence band at a position more positive than 2.5 eV *vs*. NHE. TiO_2_ (P25), with a sufficiently positive valence band potential of *ca*. 2.8 eV *vs*. NHE and superior stability, would be the best candidate catalyst. The surface of P25 has to be well-designed not only to provide active reaction sites but also to avoid over-oxidation of valuable chemicals. Metallic Pd was reported as an efficient catalytic site for benzene hydroxylation by thermal catalysis^2^, which could also enhance the charge separation^23,24^. Furthermore, isolated Cu species were reported as the active sites for benzene dissociation by forming copper-oxyl intermediates^25,26^.

In this work, sophisticated atomically Cu-coated Pd nanostructures are fabricated on P25 to decrease the energy barrier for benzene activation and avoid over-oxidation. To further avoid other by-product production, an unprecedented four-phase interfacial photocatalysis process is designed. We find that the catalyst Pd–Cu/P25 presents very high activity, and more importantly shifts the carbon selectivity towards phenol up to 92.7%, leading to the highest turnover number (TON) of over 100,000 with respect to catalytic sites of Cu on the sample of Pd_0.006_Cu_0.002_/P25.

## Results

### Photocatalytic activities

The four-phase interface (solid photocatalyst-water-benzene-oxygen) can be achieved in a suspension system, which takes advantage of (i) low solubility of benzene in water, (ii) higher solubility of phenol in benzene than in water^27^, (iii) relatively higher solubility of O_2_ in water compared with that in benzene^28^ and (iv) extreme hydrophilicity of the metal oxide P25 catalyst. During the reaction, the Cu–Pd alloy cocatalysts-decorated TiO_2_ samples were only dispersed in the water phase and drove the reaction at the interface between water and benzene where the catalyst contacted the reactant benzene and the oxidant O_2_. Following that, the synthesised phenol molecules were extracted to the benzene phase at room temperature due to their very low solubility in water, thus in situ separating the product from active oxidative species (e.g., hydroxyl and superoxide radicals) to avoid over-oxidation^29,30^. Such a nearly uniform suspension system containing these components was shown in the Supplementary Video 1 and Supplementary Fig. 1 (the left panel), in which before agitation the top layer was benzene and the bottom layer was the photocatalyst-dispersed aqueous solution. When applying moderate agitation, a uniform system has been achieved to maximise the contact between three reactants and the catalyst.

**Fig. 1:**
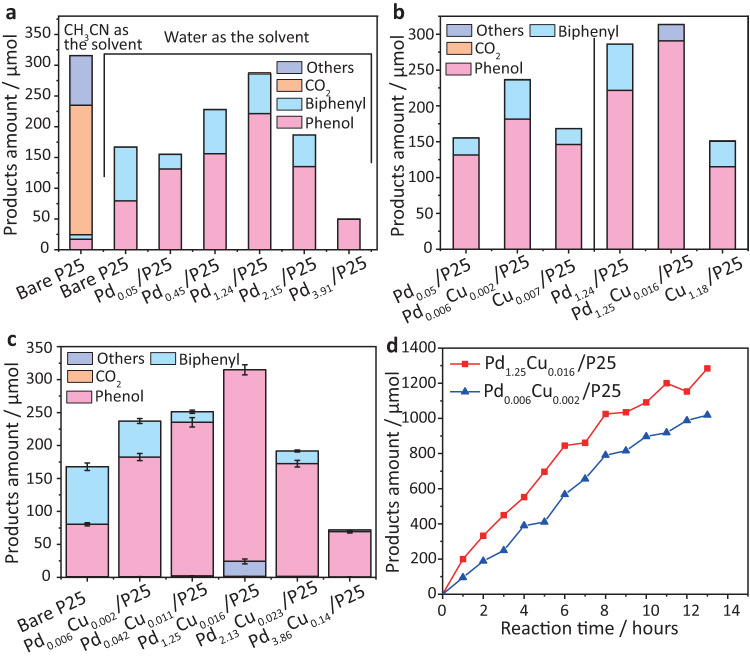
Catalytic performance of various photocatalysts. **a** The amounts of products generated in photocatalytic benzene oxidation by bare P25 and different Pd decorated P25 in 2-h in either CH_3_CN or H_2_O solution. **b** Comparison of the activity of Pd, Cu or bimetallic Pd–Cu decorated P25. **c** Optimisation of the Pd to Cu ratio, the amounts of the products were the average of six runs and the error bars were the standard deviation of six measurements (the detailed values are shown in Supplementary Table 2). **d** Phenol generation rate over Pd_0.006_Cu_0.002_/P25 (the sample with the highest TOF) and Pd_1.25_Cu_0.016_/P25 (the sample with the highest phenol yield) in the four-phase system. Reaction conditions: 30 mg photocatalysts, 10 ml water (except in the first column of (A) 10 ml CH_3_CN was used as the solvent for one control experiment), 20 ml benzene, pH = 2 adjusted by H_3_PO_4_, 365 nm LED irradiation and operated at 25 °C. Herein all cocatalyst concentration was analysed by ICP-AES.

The reactor was then purged by simulated air (20% O_2_ in N_2_) to avoid the impact of CO_2_ and sealed by a screw-type cap. Firstly the widely used reaction system was employed in which acetonitrile was used as a solvent to achieve a homogeneous reactant-solvent system and TiO_2_ was the catalyst (Fig. 1a). Then water was applied as the only solvent to form the four-phase interface system. One can see that phenol only has *ca*. 27% selectivity, with about 210 µmol CO_2_ produced when acetonitrile was used as the solvent. While replacing acetonitrile with water as the solvent, the yield of CO_2_ and other products was dramatically reduced to less than 0.2% and the yield of phenol was increased from 17 to 79 µmol. Such enhancement of phenol selectivity was due to the fast extraction of phenol by benzene from the interface of benzene and water, thus mitigating the interaction between phenol and photocatalyst that was dispersed in water and avoiding the further oxidation of phenol to CO_2_. In addition, the superiority of P25 to other representative photocatalysts (e.g., WO_3_ and g-C_3_N_4_) was investigated and shown in Supplementary Fig. 2 with the related discussion alongside.

The benzene to water ratio was then optimised over bare P25 as shown in Supplementary Fig. 3. In the absence of water, phenol and biphenyl were observed with very low production of *ca*. 1 μmol. When introducing a small amount of water (water to benzene ratio from 1:300 to 1:30), both activity and selectivity were kept almost constant, likely owing to a worse suspension of hydrophilic photocatalysts in benzene. Further increasing the water-to-benzene ratio to 1:10 resulted in a 5-times higher benzene conversion than that in the absence of water. However, the main product was biphenyl with a selectivity of 89%. When the water-to-benzene ratio rose to 1:2, the benzene conversion further increased, and the selectivity of phenol reached 40%. When more water was added to the system, the selectivity of CO_2_ in products increased from *ca.* 0.1% (water to benzene = 1:2) to 1.7% (water to benzene = 1:1) and 2.8% (water to benzene = 2:1), respectively, which was likely owing to the slower extraction of phenol from the water phase. The increase of the water-to-benzene ratio to 300:1 and 15,000:1 increased CO_2_ selectivity to nearly 100%. This is consistent with our assumption that the over-oxidation of benzene could be avoided when the products could be immediately separated from the oxidative environment and photocatalyst P25. As discussed alongside Supplementary Fig. 4, either acidic or alkaline conditions improved the phenol yield, while each facilitated different half-reactions. One was to promote oxygen reduction and the other was to promote hydroxyl radical generation. More biphenyl was also generated as a by-product under acidic conditions over bare TiO_2_, suggesting that the modification of photocatalyst played an important role in the stoichiometric generation of benzyl and hydroxyl radicals to selectively form phenol. The use of phosphoric acid to adjust the pH value to 2 presented the highest overall generation rate of phenol and biphenyl. This was likely because phosphoric acid, as a weak acid, could perform as a buffer and maintain the pH value consistently during the reaction as presented in Supplementary Table 1.

To further enhance the photocatalytic activity and the selectivity, palladium was deposited onto P25 under the optimised reaction conditions, because it was reported that Pd not only showed superior activity in the direct benzene oxidation by oxygen gas as a thermal catalyst at high temperature^2^ but also was proved to be a good charge acceptor to retard charge recombination^31,32^. As shown in Fig. 1a, even loading a tiny amount of Pd (0.005 wt%) on P25, the generated amount of phenol was increased to 170% compared to the bare P25 sample in water solution and even 7.7 times higher than that achieved in the acetonitrile solution. Furthermore, the formation of by-products was mitigated, resulting in an increase of phenol selectivity from 27% (bare sample in acetonitrile) to 73.3% (Pd_0.005_/P25 in a four-phase system). Further increasing the Pd loading amount to 0.45 wt% and 1.24 wt%, the phenol generation amount could be further enhanced. The highest phenol yield achieved over Pd_1.24_/P25 was 222 µmol, which was nearly 3 times higher than that generated over the bare P25 in water solution and 13 times higher than over bare P25 achieved in the acetonitrile solution. But the phenol selectivity was hard to be further improved and kept at *ca*. 60% when the Pd amount was over 1.24%. When the Pd loading amount further reached 2.15 wt% and 3.91 wt%, the amount of phenol generated was greatly decreased. One possible reason was attributed to the obstruction of the light absorption of P25 due to scattering^33^ or absorption^34^ by Pd nanoparticles, which was evidenced by the UV-Vis absorption measurements as shown in Supplementary Fig. 5.

It was also reported that the isolated Cu species lowered the activation energy of the C–H bond dissociation in thermal catalysis^25,26^. Thus, Cu was anchored onto Pd as another cocatalyst. As shown in Fig. 1b, when only loading Cu, both phenol and biphenyl yields over Cu_0.007_/P25 were slightly higher than that over Pd_0.005_/P25, indicating Cu could have a little better catalytic function than Pd. When co-loading both Cu and Pd on P25. Pd_0.006_Cu_0.002_/P25 showed 1.4 times higher phenol yield than that of Pd_0.005_/P25 with a selectivity of 62.2% (the left panel in Fig. 1b). These results proved a good synergy between the two cocatalysts. Next, the amount of Cu–Pd bimetallic particles was optimised as shown in Fig. 1c. When increasing the amount of both Pd and Cu in the bimetallic clusters, the selectivity towards phenol was greatly enhanced to around 90% while the phenol generation amount was between 1.3–1.5 times higher than the corresponding sample decorated by Pd only, indicating a tiny amount of Cu (0.01–0.02 wt%) dramatically improved both the yield and selectivity of phenol production. The sample Pd_1.25_Cu_0.016_/P25 even presented a selectivity of 92.6% and yield of phenol of 4833 µmol g^−1^ h^−1^ with no detectable biphenyl and a small number of other products (e.g. benzoquinone and acetone). The right panel of Fig. 1b and Table 1 further confirmed that the activity and selectivity over the Pd–Cu co-decorated samples were very different from the samples modified by either Pd or Cu, indicating the synergy effect of Pd and Cu in the activity and selectivity control, although Cu seems to play a bigger role in selectivity.

**Table 1:** TON of Pd, Cu or bimetallic Pd–Cu decorated P25 and bare P25 during a 2-h test

Catalysts	Phenol generation amount (μmol)	TON
Bare P25	79.51	0.11
Pd_0.05_/P25	131.27	928
Pd_0.006_Cu_0.002_/P25	181.39	19,347
Cu_0.007_/P25	146.15	4454
Pd_1.24_/P25	221.53	63
Pd_1.25_Cu_0.016_/P25	290.70	3876
Cu_1.18_/P25	115.10	21

As shown in Supplementary Table 3, most of the photocatalytic processes had to employ environmentally unfriendly, strong and costly H_2_O_2_ rather than O_2_ as the oxidant to achieve the high activity. The high yield of phenol when utilising H_2_O_2_ was due to the easy process of hydroxyl radicals production by reduction of H_2_O_2_, while oxidation of water for hydroxyl radicals production is much more challenging and scientifically more significant^35^. Using oxygen as the oxidant was also very challenging, resulting in rather low activity^21,36,37^. Compared with those using O_2_ as the oxygen source (thermal catalysis, No 10, and photocatalysis, No 7-10 in Supplementary Table 3), our photocatalytic system exhibited the highest yield of phenol, e.g., at least 25 times higher than previous benchmark work, e.g., 0.19 mmol/g/h over Bi_2_WO_6_/CdWO_4_^21^, 0.00625 mmol/g/h over TiO_2_@MCF/CH_3_/UV^36^. Even compared with the benchmarks achieved in thermal catalysis, our new catalyst using air as the oxidant operating at room temperature showed both comparable selectivity and yield of phenol to that using H_2_O_2_ as the oxidant operated at high temperature in thermal catalytic processes^4,8–11,38^.

The stability tests over three representative samples, Bare P25 (Supplementary Fig. 6), Pd_0.006_Cu_0.002_/P25 (the sample with the highest TOF) and Pd_1.25_Cu_0.016_/P25 (the sample with the highest phenol yield), were then conducted as shown in Fig. 1d. All samples showed an almost linear increase of phenol generation for 12 h, which proved the effectiveness of the multi-phasic interfacial reaction strategy in the colloid system. We also analysed both benzene and water solution by GC-MS and found out the product phenol was almost entirely dissolved in benzene rather than in water. Thus, phenol could be efficiently extracted to the benzene phase while the hydrophilic photocatalysts and oxidant O_2_ were dispersed in the water phase. Over-oxidation could be mitigated during the generation and collection of phenol. To further prove the concept of the four-phase system over Pd_1.24_Cu_0.016_/P25, the control experiment in a homogeneous reactant-solvent system was conducted, which was consistent with our analysis that the valuable products were more active than benzene and easier to be further oxidised when homogeneously mixed with the photocatalysts and O_2_. As shown in Supplementary Figs. 7 and 8, the selectivity towards phenol in the homogeneous reactant-solvent system dissolved in acetonitrile was 20 times lower than in the four-phase system. It must be noted that acetonitrile was also able to be activated in such a reaction system to form coupling products (e.g., butanedinitrile and phenylacetonitrile) and oxidised products (e.g., acetamide and oxalamide). Therefore, we suggest that the utilisation of acetonitrile for the activation of stable molecules requires prudential consideration. It was also noteworthy that the selectivity towards CO_2_ decreased from 67% over bare P25 to 50% over Pd_1.24_Cu_0.016_/P25 in the homogeneous system, indicating the capability to mitigate the further oxidation of phenol over the decorated sample.

Overall, based on the 12-h test, the highest turnover number (TON) of over 100,000 was achieved by Pd_0.006_Cu_0.002_/P25, where Cu formed the shell over Pd, and Cu species were believed to be the active reaction sites as discussed later. Namely, a TOF of *ca*. 2.7 s^−1^ was obtained. TOF of *ca*. 1 s^−1^ is the golden rule for a heterogeneous catalyst in any liquid reactions^39^ and the previous benchmark result for benzene oxidation to phenol by O_2_ using thermal catalysis represented a TOF 0.04 s^−1^ ^2^ and using photocatalysis a TOF of 10^−4^ s^−1^ ^21^.

### Catalyst characterisation

The chemical states of the Pd and Cu species before and after the 12-h reaction were studied by XPS (Supplementary Figs. 9, 10; Supplementary Tables 4, 5). 43.6% of oxidised Pd species can be observed on the fresh sample, which almost remains unchanged (43.2%) after the reaction (Supplementary Table 4). However, the position of Pd(II) peak shifts 0.2 eV more positively after a 12-h reaction, which is consistent with the later proposed reaction mechanism of Pd species as the hole acceptor. The fitting results also suggest the presence of Cu(II), 41.4% Cu(II) species before the reaction and 41.6% Cu(II) species after the reaction (Supplementary Table 5). The presence of oxidised Pd or Cu species is not surprising taking the preparation method into consideration. A photo-deposition method was used to load PdCu on P25. After that, they may start to exert their function as the hole acceptor in the suspension under light irradiation. Furthermore, the extent of oxidised Cu relies on the exposure time in the air and a longer time may lead to more surface Cu oxidation. This is common in PdCu nanoalloy particles since Cu on the surface of PdCu nanoalloy can be oxidised more easily according to previous reports^40,41^.

**Fig. 2:**
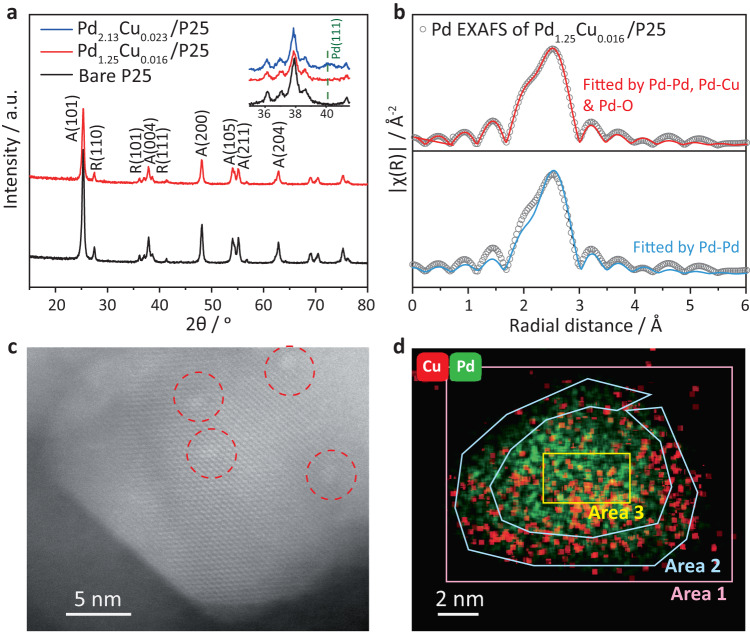
Physical observations of the synthesised catalysts. **a** PXRD patterns of Pd_1.25_Cu_0.016_/P25, bare P25 and zoomed PXRD patterns of Pd_2.13_Cu_0.023_/P25, Pd_1.25_Cu_0.016_/P25 and bare P25; **b** Pd *k*-edge EXAFS spectra of Pd_1.25_Cu_0.016_/P25, fitting by considering Pd–Pd only (bottom) and all of Pd–Pd, Pd–O and Pd–Cu (top); **c** HAADF-STEM of Pd_1.25_Cu_0.016_/P25. The light spots highlighted by red circles represent Pd–Cu particles. **d** EDX elements mapping of a representative Pd–Cu particle indicating Cu species were the outmost in Pd–Cu particles.

The actual Pd and Cu amount loaded was confirmed by inductively coupled plasma atomic emission spectroscopy (ICP-AES) and named as Pd_x_Cu_y_/P25, where x% and y% denoted the weight percentage of Pd and Cu, respectively. The crystal structures of the as-synthesised samples were first investigated by powder X-ray diffraction (PXRD). As shown in Fig. 2a (three representative samples) and Supplementary Fig. 11 (all samples), the P25 diffraction patterns were not affected by the Pd and Cu deposition^42^. When increasing the Pd amount from 1.25 wt% to 2.13 wt% a small peak located at *ca*. 2*θ* = 40° was observed as shown in the insert of Fig. 2a. This could be assigned as the Pd(111), indicating the existence of metallic Pd species (JCPDS 87-0638)^43,44^. However, Cu species showed no obvious profile in the XRD pattern, likely owing to the very low concentration. The chemical states of the Pd species were then investigated by Extended X-ray absorption fine structure (EXAFS) spectra (Fig. 2b and Supplementary Fig. 12)^45^. It further confirmed the existence of metallic Pd species but the use of only the Pd–Pd shell was not sufficient for an acceptable fitting (Fig. 2b bottom panel)^46^. When considering the contribution of Pd–Pd, Pd–Cu and Pd–O bonds together in the first shell, as shown in Fig. 2b top panel and Supplementary Table 6, a much better fitting was achieved, indicating there was a strong interaction between Pd and O atoms besides Pd–Pd and Pd–Cu species^43,47^. This was consistent with the XPS analysis above. The fitting results listed in Supplementary Table 4 also showed that the Pd–Pd shell had a bond length of 2.73 ± 0.01 Å that was close to the Pd foil of 2.75 Å^46^. The bond length of Pd–Cu was slightly shorter than Pd–Pd with a small coordination number of 0.95 ± 0.01 indicating Cu species were atomically dispersed on Pd particles. A Pd–O interaction was also observed with a distance of 1.99 ± 0.05 Å, which was slightly larger than the Ti–O bond length of 1.93 Å in anatase^48^, suggesting Pd species were likely decorated on P25 via Pd–O interaction. To further confirm the interaction between Pd and TiO_2_, Ti K-edge XAFS was also carried out. The XANES results were shown in Supplementary Fig. 13, the edge position of samples with and without Pd/Cu decoration showed no obvious change, indicating a similar chemical state of Ti atoms in the bare and decorated samples. The pre-edge shape of the Pd–Cu decorated sample also remained very similar to the shape of the bare sample, suggesting a similar symmetry of the crystal structure due to the low concentration of Pd–Cu loaded. However, the Fourier transformed EXAFS results as shown in Supplementary Fig. 14 represented that the first shell peak shifted to the higher value, indicating a longer Ti–O distance. Taking into account the XRD results as shown in Fig. 2a, there was no obvious phase change of the samples with and without cocatalyst decoration. Therefore, the longer Ti–O distance was likely owing to the interaction between O atoms and Pd/Cu alloy sites. Consistently, the second shell Ti–Ti (Pd and/or Cu) distance became shorter, which also suggested the chemical binding between TiO_2_ and Pd/Cu in part. The Raman spectra as shown in Supplementary Fig. 15 also presented an obvious blueshift of the *E*_g_ peak located at *ca*. 144 cm^−1^. This peak is mainly attributed to the symmetric stretching vibration of O–Ti–O in TiO_2_. The higher vibrational wavenumbers (blue shift) indicated a closely packed TiO_2_ due to compressive stress on the first several layer atoms of TiO_2_ by Pd–Cu decoration^49,50^, which was in part consistent with the shorter second shell Ti–Ti distance suggested by EXAFS.

The distribution of Pd and Cu species was then studied by High-angle annular dark-field scanning transmission electron microscopy (HAADF-STEM). As shown in Fig. 2c and Supplementary Fig. 16A, the cocatalyst Pd_1.25_Cu_0.016_ had small particles, mainly of *ca*. 2 nm with a few larger than *5* nm (labelled in Supplementary Fig. 16A). The Energy Dispersive X-Ray Analysis (EDX) as shown in Supplementary Figs. 16B and C and Supplementary Table 7 confirmed that these particles were composed of Cu and Pd. Figure 2d and Supplementary Fig. 17 further suggested that the Cu to Pd ratio was much higher on the edge than that in the core of the Cu–Pd particles. Such results were in good accordance with the fact that the Cu to Pd ratios obtained by XPS, a surface-sensitive technique, were much higher (Cu:Pd = 1.25:1) in comparison with the ratio obtained by ICP-AES (Cu:Pd = 0.02:1), which is one of the best techniques to obtain the bulk composition, as shown in Supplementary Table 8. This clearly indicated that Cu was mainly located on the surface of the Pd–Cu alloy. Supplementary Fig. 18 also showed that even in the trace amount of Cu and Pd co-loaded P25 (the catalyst Pd_0.006_Cu_0.002_/P25), the Pd–Cu were still in the form of near core-shell clusters with a size of *ca*. 1 nm.

### Mechanism investigation

**Fig. 3:**
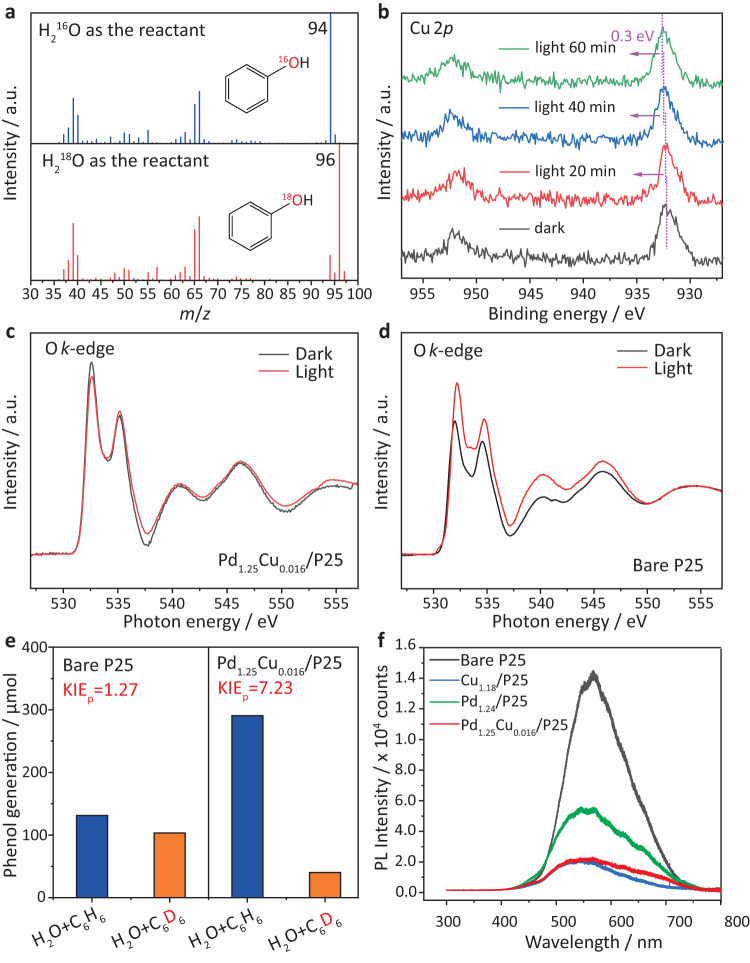
Fundamental characterisations of the catalytic process. **a** Mass spectra of generated phenol using oxygen isotopic labelled water as the reactant over Pd_1.25_Cu_0.016_/P25; **b** in situ Cu 2*p* XPS spectra of Pd_1.25_Cu_0.016_/P25 under the light on/off conditions; **c** in situ O K-edge NEXAFS spectra of Pd_1.25_Cu_0.016_/P25 in the dark/light irradiation condition; **d** in situ O K-edge NEXAFS spectra of P25 in the dark/light irradiation condition. **e** Kinetic isotopic effects of phenol generation using deuterated benzene as reactants over Pd_1.25_Cu_0.016_/P25 and bare P25 at pH = 2. **f** Photoluminescence spectra excited by 365 nm laser of cocatalysts-decorated P25.

**Fig. 4:**
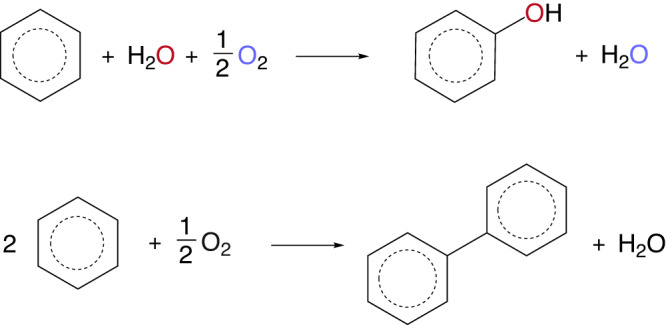
The overall chemical reaction equations. Equation 1 is the oxidation of benzene to phenol and Equation 2 is the oxidative coupling of benzene to biphenyl.

The reaction mechanism was then investigated by isotopic labelling. Oxygen-labelled water was first employed in the reaction under identical experimental conditions, as shown in Fig. 3a, to confirm the oxygen source in the generated phenol molecules. When using the H_2_^16^O as the reactant and solvent, all peaks in the mass spectrum were able to be identified by the standard phenol spectrum in the database (NIST 133909). When replacing water with the ^18^O-labelled water (H_2_^18^O), the strongest peak at *m/z* = 94 shifted to *m/z* = 96, indicating most of the generated phenol was labelled by ^18^O. Therefore, the main oxygen source in the generated phenol was from the oxidised water rather than the reduced oxygen species. As shown in Supplementary Table 9, the produced ^18^O and ^16^O labelled phenol ratios remained almost constant with a ratio of *ca*. 5.5 no matter how long the reaction took place, further indicating that the oxygen in the product of phenol was mainly from water, rather than O_2_ gas. A control experiment was also conducted to confirm no oxygen exchange between the generated phenol and ^18^O labelled water. As shown in Supplementary Fig. 19, there were no obvious changes in phenol mass spectra after 2 h, suggesting no detectable oxygen exchange between phenol and water. To investigate the function of the O_2_ gas, the reactions were carried out in an inert argon atmosphere and compared with that in the air. As shown in Supplementary Fig. 20, the phenol yield was dramatically decreased by *ca*. 15 times in the argon atmosphere. Furthermore, hydrogen gas was produced as a by-product while its generation rate was much lower than the stoichiometric ratio, which supported the hypothesis that the presence of O_2_ could accelerate the reaction rate with protons, providing a downhill path to regenerate the proton poisoned active sites on P25 to form water instead of H_2_ evolution. An ^18^O_2_ isotope experiment was further carried out to verify this process, as shown in Supplementary Fig. 21 with a discussion alongside. The overall chemical reaction equations were shown in Equations 1 and 2 in Fig. 4.

The charge transfer pathways of photo-induced charge carriers were evaluated by in situ XPS in the presence and absence of Xenon light irradiation. As shown in Supplementary Fig. 22, under light irradiation, there was no obvious shift of Pd 3*d* peak position, while the peaks of Cu 2*p* gradually shifted to the higher binding energy of 0.3 eV after 1-h irradiation as shown in Fig. 3b. Therefore, Pd–Cu species very likely worked as a hole acceptor, and the photoholes transferred from the valence band of TiO_2_ to the outmost Cu species via Pd, which also indicated that Cu species were the active sites for the oxidation process. To further confirm PdCu as the hole acceptor, the in situ near-edge X-ray absorption fine structure (NEXAFS) spectra were carried out, as shown in Fig. 3c, d. The O K-edge NEXAFS feature of TiO_2_ arises from the transition from the occupied O 1s orbital to the O 2p orbital, whose intensity is inversely proportional to the density of occupied O 2p states. In other words, more unoccupied O 2p states can allow more electrons to transition from O 1s orbital. The valence band (VB) of TiO_2_ consists of the O 2p orbital, thus the in situ O K-edge NEXAFS spectra can provide unambiguous information about the photoholes on the VB. Under light irradiation, the electron on the VB of TiO_2_ is excited to the CB of TiO_2_, leaving holes on the VB, which can be considered as creating more unoccupied O 2p states. Thus, the intensity of O K-edge NEXAFS spectra should increase, as shown on Bare P25 in Fig. 3d^51^. In contrast, almost no change of intensity can be observed after the introduction of PdCu (Fig. 3c), suggesting that the holes can transfer to PdCu or PdCu can donate electrons to fill the unoccupied O 2p states on TiO_2_. This result is consistent with our in situ XPS results, further consolidating PdCu as the hole acceptor. To further clarify the reaction mechanism, DFT calculations were carried out. A tetrahedral cluster with three palladium atoms and one copper atom was put on the TiO_2_ anatase (101) surface, as shown in Supplementary Fig. 23, with the structure based on a Pd_4_ cluster on anatase (101) from an earlier computational study^52^. Charges on atoms were calculated using Mulliken population analysis. Mulliken charges of all three Pd atoms were 0.08−0.14 e larger than the formal nuclear charge, indicating that Pd atoms of the cluster gained electron density, as shown in Supplementary Table 10: the overall electron gain for the three Pd atoms was −0.346. In contrast, the Cu atom charge in the cluster was 0.436 smaller than the formal charge of a Cu atom, indicating that the Cu atom lost electron density. Thus, the overall charge of the adsorbed Pd_3_Cu_1_ cluster was 0.09 e more positive than an isolated cluster, indicating that the cluster as a whole donated electron density to TiO_2_. This suggested that the cluster would work as a hole acceptor, and the hole could transfer from TiO_2_ to Pd and then to the outermost Cu, which is consistent with the in situ XPS and NEXAFS. In addition to the above ground-state charge transfer, the charge density after the excitation was also modelled as shown in Supplementary Fig. 24. Photoexcitation resulted in a transfer of photoelectrons from PdCu to the conduction band of TiO_2_. Namely, the density of states showed that the highest occupied states were localised on Pd and Cu, while the lowest unoccupied states were on TiO_2_. This suggested that upon photoexcitation of TiO_2_, photoholes were able to go to PdCu as its photoelectrons were excited to the conduction band of TiO_2_. Therefore, our computational results showed that photoexcitation increased the number of holes on PdCu nanoparticles. Briefly, the outermost Cu atoms were the ultimate hole acceptors in the Cu–Pd/TiO_2_ system and Pd clusters helped the charge transfer. The Cu–Pd synergy was essential in enhancing the capability of holes separation from electrons and transfer. The binding energies of water on Pd and Pd–Cu clusters on TiO_2_ were also compared as shown in Supplementary Table 11. The water molecule adsorbed more strongly on the Cu atom of Pd_3_Cu_1_ (−0.64 eV) than on an outermost Pd atom of a Pd_4_ cluster (−0.46 eV). Therefore, the Cu species at the shell of the alloy could enhance the interaction with water to generate more hydroxyl radicals, favoring phenol production over other products. Such modelling results were confirmed by O 1*s* XPS spectra as shown in Supplementary Fig. 25. The Pd_1.25_Cu_0.016_/P25 surface had the strongest shoulder peak located at 532 eV which was assigned as the adsorbed OH species. Therefore, Pd_1.25_Cu_0.016_/P25 was estimated to have the strongest capability for water adsorption. The capability for the oxidation of water and benzene was also experimentally confirmed as shown in Supplementary Figs. 26–28^53–55^. The generated photoholes accumulated on Cu sites were positive enough to drive the water oxidation to produce ·OH radicals or even to generate O_2_ although the latter is much more difficult than the former due to the four-hole chemistry required. The potential of ·OH radicals production from water E^0^(·OH/H_2_O) is 2.8 eV (*vs*. NHE), which is more positive than the oxidation potential of benzene of 2.5 eV (*vs*. NHE). Therefore, the photoholes on PdCu should be able to oxidise benzene molecules via water oxidation.

**Fig. 5:**
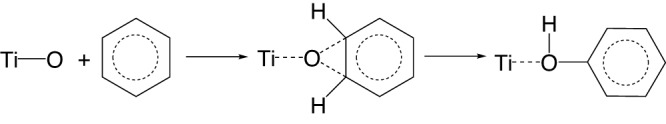
The reaction pathway via an epoxy-type adsorbed intermediate on TiO_2_.

To further evaluate the rate-determining step (RDS) of this reaction, the kinetic isotope effect (KIE) was studied by deuterated reactants. As shown in Supplementary Figs. 29, when using deuterated water as the reactant, the *k*_H_/*k*_D_ ratios presented on bare P25 and Pd–Cu/P25 were 1.22 and 1.19, respectively. The values of KIE were very close to 1, indicating that the activation of hydroxyl species was not the rate-determining step. On the contrary, as shown in Fig. 3e, the deuteration of benzene resulted in the phenol generation amount decreased to *ca*. 14% compared to untreated benzene as the reactant, over Pd_1.25_Cu_0.016_/P25 (the right panel of Fig. 3e). However, the generated phenol only fell to *ca*. 79% when using deuterated benzene over bare P25 (the left panel of Fig. 3e). Thus, the calculated KIE factors were 7.23 for Pd_1.25_Cu_0.016_/P25 and 1.27 over the bare P25 sample, respectively. Such differences in KIE were also reported in thermal catalysis. Two reaction paths for C–H activation with two types of rate-determining steps were proposed^56^. As shown in Equation 3 in Fig. 5, one of the reaction mechanisms was via an epoxy-type adsorbed intermediate, where lattice oxygen atoms were involved in the reaction to epoxidate the aromatic ring. When the C–H breaking rate was faster than the epoxidation process, the overall reaction rate was determined by the benzene adsorption rather than the C–H breaking. Therefore, the deuteration of the reactant only showed a slight impact on the reaction kinetics, resulting in a KIE close to 1 which was typically defined as the normal secondary KIE.

**Fig. 6:**
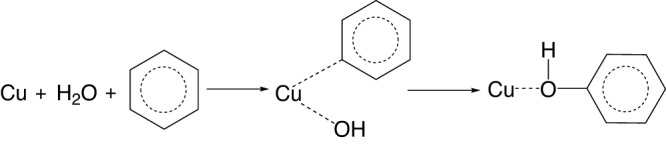
The reaction pathway determined by C–H breaking on Cu species.

However, on the Cu–Pd decorated surface, there were no lattice oxygen atoms at the outmost shell Cu species. Thus, a different reaction mechanism determined by the C–H breaking could be assumed as shown in Equation 4 in Fig. 6. Both water and benzene were dissociated on the Cu active sites and then formed phenol. Therefore, an obvious isotope effect for the C–H breaking was observed (also known as the primary KIE).

To further investigate the rate-determining step, several photocatalytic reactions were carried out in the presence of a hole scavenger (ammonium oxalate, AO) and hydroxyl radical scavenger (isopropanol, IPA), respectively (Supplementary Fig. 30)^57,58^. It should be noted that the introduction of a hole scavenger greatly limits the generation of phenol, which is only *ca.* 4% of that achieved under the original condition. This suggests the indispensable role of photoholes in benzene activation. In contrast, almost no change in phenol production can be observed with the addition of hydroxyl radicals scavenger IPA. Two conclusions can be drawn from this study: (i) the benzene molecule is directly activated by photoholes on Cu rather than the indirect activation pathway by hydroxyl radicals formed by water and photoholes; (ii) this direct activation step instead of the water oxidation is the rate-determining step in the whole catalytic cycle. This is also consistent with the KIE effect, in which the deuterated water shows no KIE effect while the primary KIE effect can be observed with the deuterated benzene on PdCu/P25. The enhancement of water activation by Cu was also proved by DFT calculation and other experiments as mentioned above, which allows the existence of sufficient hydroxyl radicals to couple with benzyl radicals after formation. Thus, the addition of a tiny amount of IPA can hardly affect the phenol yield rate.

The above results suggested that Cu species directly contributed to the activation of benzene and the active sites which were different from the bare sample. According to the EXAFS results, as shown in Fig. 2b and Supplementary Table 4, the Pd–Cu coordination number was close to 1, indicating that Cu species were highly dispersed on Pd particles. Thus, the distance between two adsorbed benzenes on Cu species was much larger than on the surface of bare P25. Therefore, the recombination of two adsorbed benzene molecules to form biphenyl was much more difficult on the Cu decorated surface than on the bare P25 surface.

Supplementary Fig. 31 presented the light absorption spectra of the catalyst. All cocatalyst decorated catalysts showed a slight redshift of the adsorption edges, which was likely due to the d-band transition of metal species^34^. Thus, a control experiment was next conducted to exclude the contribution from the possible d-band excitation of Pd–Cu particles (see the discussion along with Supplementary 31)^34^. Furthermore, both Pd_1.24_/P25 and Pd_1.25_Cu_0.016_/P25 presented the tail adsorption in the visible region, which resulted from the scattering of Pd nanoparticles^32,59^.

The photoluminescence (PL) measurements of these samples were undertaken when excited by a 325 nm laser. As shown in Fig. 3e the bare sample showed the highest intensity, which was more than double of Pd_1.24_/P25 and more than 7 times higher than Cu_1.18_/P25 and Pd_1.24_Cu_0.016_/P25. As the light absorption was similar at 325 nm as shown in Supplementary Fig. 31 for all four samples, the PL intensity was mainly associated with the recombination rate between photogenerated electrons and holes. It could be estimated that the deposited cocatalysts greatly enhanced the charge transfer and separation in the photocatalysts. The PL measurements were also conducted under 365 nm laser irradiation as shown in Supplementary Fig. 32. Similarly, the signal intensity of Pd_1.24_Cu_0.016_/P25 was one-third of the intensity over the bare sample.

**Fig. 7:**
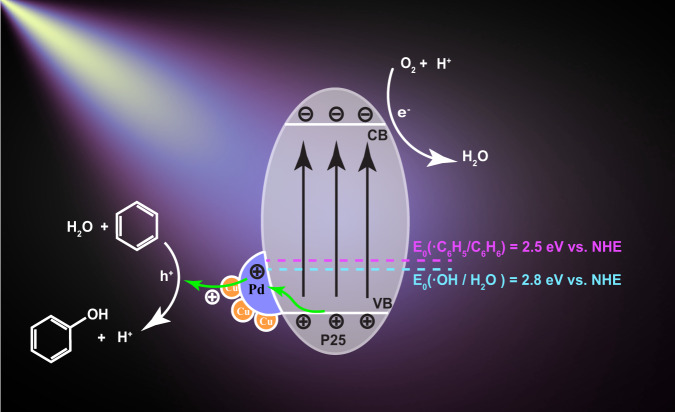
Schematic of charge transfer during selective benzene oxidation to phenol over Pd–Cu/P25. (VB is the top of the valence band and CB is the bottom of the conduction band). Both OH radical production potential and benzene oxidation potential are indicated.

Therefore, a typical reaction mechanism for benzene conversion to phenol over Pd–Cu/P25 in one step was proposed as Fig. 7. In general, incident photons first excited the electrons from the valence band of P25 to the conduction band while holes were left in the valence band. On the bare P25, the photogenerated holes were able to react with benzene over the oxygen sites to form epoxy-type adsorbed intermediates. The desorption of an intermediate via Ti–O cleavage resulted in the formation of phenol, while the coupling of two intermediates led to the generation of biphenyl. On the contrary, over Pd–Cu/P25, photoholes transferred from the valence band of P25 to Pd particles and then to the outmost atomically dispersed Cu species. The Cu sites presented the strong capability for water binding and oxidation to form hydroxyl radicals as suggested by both experiments and DFT calculations. Cu was also able to activate benzene to form benzyl radicals at the same site indicated by the KIE measurements. Thus, phenol was generated via the coupling between hydroxyl and benzyl radicals. As the atomic Cu sites were isolated and highly dispersed, thus the recombination of two benzyls was much more difficult than on the bare P25. This is not to say Pd can be neglected. Without Pd, the decoration of sole Cu species on P25 leads to moderate phenol production, less than half of the optimised sample Pd_1.25_Cu_0.016_/P25 (Fig. 1b). The increase in the conversion rate indicates more available photoholes to participate in the reaction. The important role of Pd is to retard the charge recombination and promote charge separation to facilitate the reaction. From the in situ XPS results (Supplementary Fig. 22 and Fig. 3b), the Pd act as an important media to efficiently extract the photoholes from TiO_2_ and transport them to the outmost Cu to drive the reaction. Therefore, such atomically Cu-coated Pd nanoclusters were significant as they improved the charge transfer and separation, decreased the activation energy of oxidation of both water and benzene, thus opening a new route on the direct formation of phenol with very high selectivity.

## Discussion

In summary, atomically Cu-coated Pd nanoparticles have been successfully anchored on P25. The optimised sample presented the highest selectivity of 92.6% towards phenol with a generation rate of 4833 µmol g^−1^ h^−1^. In parallel, the highest TON of over 100,000 (or TOF of 2.7 s^−1^) has been achieved by Pd_0.006_Cu_0.002_/P25 (relative to the outmost Cu species). Based on the structural characterisation, isotopic labelling, kinetic analysis, spectroscopic measurements and DFT calculations, the superior activity of Pd–Cu co-decorated samples could be ascribed to the efficient hole transfer from P25 to the Pd–Cu nanostructures. Water as a promoter was also proved to be crucial for the high selectivity to phenol. More importantly, the Pd–Cu nanostructures were able to control the selectivity towards phenol rather than biphenyl by opening a new low-energy pathway for the C–H bond cleavage and avoiding the dimerization of phenyl species. Furthermore, the optimised interfacial reaction strategy in a suspension system can generate an oxidant-lean environment that spontaneously protects the high-value products from over-oxidation. In addition, this reaction has strong potential to be readily scaled up in a commercially available continuous stirred tank reactor. Such novel design protocols for both photocatalysts and reaction systems could open an economical pathway for the selective conversion of highly symmetric molecules.

## Methods

### Fabrication of photocatalysts

A highly reproducible photo-deposition method was used to prepare the decorated P25 photocatalysts. In a typical run, a certain amount of palladium(II) chloride (Sigma-Aldrich, ReagentPlus®, 99%) and copper(II) acetate (Sigma-Aldrich, 98%) were dissolved in 100 mL deionised water. The solution was transferred to a 250 mL quartz reactor and then 500 mg P25 was added. The reactor was sonicated for 15 min to make the suspension uniform. Then the reactor was sealed by a quartz window and purged by argon for 15 min. After purging, the suspension was irradiated by a full arc 300 W Xenon lamp (Newport) with vigorous stirring (700 rpm). After 8-h Xenon lamp irradiation, the suspension was kept stirred in the dark for another 12 h. Then, the samples were separated by a centrifuge and washed with deionised water three times. The samples were then dried in an oven at 70 °C for 24 h. After hand grinding, the samples were then stored for characterisation and activity testing. To label the composition of the samples, 100 mg of the synthesised sample was dissolved in aqua regia and then diluted to 50 mL by water. The solution was quantified by a Varian 720 ICP-AES with axial configuration. The measured Pd weight percentage was denoted x% and the Cu weight percentage y%. Then the sample was labelled as Pd_x_Cu_y_/P25. The structural properties of bare P25 and the optimised PdCu/P25 are provided in Supplementary Fig. 33 and Supplementary Table 12.

### Characterisation

PXRD measurements were taken using a Stoe StadiP diffractometer (wavelength 0.071 nm) in London, UK. The 2θ value in the manuscript was converted to the distance values using the copper target wavelength by Bragg’s law for an easy comparison of published results. XAFS spectra of the samples were collected at SSRF (Shanghai, China) in fluorescence mode. The EXAFS oscillation was fitted according to a back-scattering equation, using FEFF models generated from the crystal structure of metallic palladium (space group Fm3m) and anatase (space group I4_1_/amd)^46^. Near-edge X-ray absorption fine structure (NEXAFS) spectra were measured at photoelectron spectroscopy end-station of National Synchrotron Radiation Laboratory of China. XPS was performed on a Thermo Scientific XPS K-alpha machine using monochromatic Al-Kα radiation in London, UK. Survey scans were collected in the range of 0–1100 eV (binding energy) at a pass energy of 160 eV. Higher-resolution scans were recorded for the main core lines at a pass energy of 20 eV. The results were fitted by CasaXPS software. An in-depth chemical analysis of the Pd–Cu immobilised on P25 was performed using a double Cs aberration-corrected FEI Titan^3^ Themis 60–300 in Cádiz, Spain. This microscope was operated at 200 kV equipped with an X-FEG gun, a monochromator, and a high-efficiency XEDS ChemiSTEM. The latter device consists of a 4-windowless SDD detector and in combination with the scanning transmission electron microscope mode, allowing to perform accurate XEDS mapping. These experiments were carried out using a beam current of 100 pA and a dwelling time per pixel of 50 μs. To improve the visual quality of the elemental maps, these were filtered using a Gaussian blur of 1 using Velox software. This software was also used for the quantitative analysis. HR-STEM imaging was performed using a high-angle annular dark-field (HAADF) detector with a camera length of 11.5 cm in Cádiz, Spain. Due to the very small content of Cu, a study of the effect of the holder configuration and the grid composition was performed using the Pd_1.24_/P25 sample. The results of this study were shown in Supplementary Fig. 31, which clearly showed that using the analytical double-tilted holder with a Mo clip and a C-coated gold grid, no misleading Cu signal can be observed. UV-Vis absorption spectra were obtained on an Agilent Cary 5000 UV-Vis-NIR spectrophotometer fitted with an integrating sphere in London, UK. Reflectance measurements were performed on powdered samples using a standard barium sulphate powder as a reference. The reflection measurements were converted to the absorption spectra using Kubelka–Mulk transformation. Photoluminescence spectra were observed on a Renishaw inVia Raman microscope in London, UK, using a 3.8 eV excitation laser and a wavelength range of 200–800 nm. In situ XPS was performed on a ULVAC-PHI-PHI 5000 VersaProbe III using a 300 W Xenon lamp as the light source. The structural properties (e.g., specific surface area, pore volume) was investigated on Multi-BET QUADRASORB evo 4 BET Stations, Austria.

### Photocatalytic activity test

The photocatalytic activity measurements of benzene conversion were conducted in a multi-channel photocatalytic reaction system (Beijing Perfectlight PCX50B, 365 nm LED). The reactor volume was 50 mL and the magnetic stir bar was suspended at the medium height of the reactor with a stirring rate of 500 rpm. The reaction temperature was controlled by a water circulator at 25 °C. The product selectivity was calculated based on all detected products as the reactant benzene was solvent and was present in excess.

In a typical test, the pH of deionised water was first adjusted by phosphoric acid (Alfa Aesar, 85% in aqueous solution) to 2, then 30 mg catalyst was suspended and subsequently sonicated in 10 mL of the acidic solution. 20 mL of benzene (Sigma-Aldrich, 99.8%) was then added to the suspension. The reactor was sealed by a cap with PETF o-ring and septa and purged by simulated air (20% oxygen in nitrogen, BOC) for 10 min. A Shimadzu GCMS-QP2010 equipped with a 30 m Rtx 502.2 column and helium as a carrier gas was used to analyse the products. The isotopically labelled reactions were carried out in a 5 mL reactor with 2 ml benzene, 1 mL water and 5 mg photocatalyst. All the other conditions were the same as the activity test reaction.

Turn-over-number (TON) was calculated by the following equation, ${{{{{\rm{TON}}}}}}=\frac{{{{{{\rm{moles}}}}}} \,{\rm {of}} \,{\rm {phenol}} \,{\rm {yield}}}{{{{{{\rm{moles}}}}}} {\rm {of}} {\rm {Cu}} {\rm {atoms}}}$. For instance, to calculate the TON of Pd_1.25_Cu_0.016_/P25 during a 12-h test as shown in Fig. 1d,

R2.1 $${{{{{\rm{Moles}}}}}}\; {{{{{\rm{of}}}}}}\; {{{{{\rm{phenol}}}}}}\; {{{{{\rm{yield}}}}}}=1284.11\,{{\upmu }}{{{{{\rm{mol}}}}}}$$

R2.2 $${{{{{\rm{Catalysts}}}}}}\; {{{{{\rm{amount}}}}}}=30\,{{{{{\rm{mg}}}}}}$$

R2.3 $${{{{{\rm{Cu}}}}}}\; {{{{{\rm{concentration}}}}}}=0.016\,{{{{{\rm{wt}}}}}}\%$$

R2.4 $${{{{{\rm{Cu}}}}}}\; {{{{{\rm{molar}}}}}}\; {{{{{\rm{amount}}}}}} 	= \frac{{{{{{\rm{Catalysts}}}}}}\; {{{{{\rm{amount}}}}}}\times {{{{{\rm{Cu}}}}}}\; {{{{{\rm{concentration}}}}}}}{{{{{{\rm{The}}}}}}\; {{{{{\rm{atomic}}}}}}\; {{{{{\rm{weight}}}}}}\; {{{{{\rm{of}}}}}}\; {{{{{\rm{Cu}}}}}}}\\ 	=\frac{30\,{{{{{\rm{mg}}}}}}\times 0.016\,{{{{{\rm{wt}}}}}}\%}{64}\\ 	=7.5\times {10}^{-2}\,{{\upmu }}{{{{{\rm{mol}}}}}}$$

R2.5 $${{{{{\rm{TON}}}}}} 	=\frac{{{{{{\rm{moles}}}}}}\; {{{{{\rm{of}}}}}}\; {{{{{\rm{phenol}}}}}}\; {{{{{\rm{yield}}}}}}}{{{{{{\rm{moles}}}}}}\; {{{{{\rm{of}}}}}}\; {{{{{\rm{Cu}}}}}}\; {{{{{\rm{atoms}}}}}}}\\ 	=\frac{1284.11\,{{\upmu }}{{{{{\rm{mol}}}}}}}{7.5\times {10}^{-2}\,{{\upmu }}{{{{{\rm{mol}}}}}}}\\ 	=17121$$

Kinetic isotope effect (KIE) measurements were carried out under the identical reaction conditions. To calculate the KIE the following equation was applied:

R2.6 $${{{{{\mathrm{KIE}}}}}}_{{{{{\mathrm{phenol}}}}}}=\frac{{{{{{\mathrm{Amount}}}}}}\; {{{{{\mathrm{of}}}}}} {{{{{\mathrm{generated}}}}}} \,{{{{{\mathrm{phenol}}}}}} \,{{{{{\mathrm{with}}}}}}\,\,{{{{{\rm{C}}}}}}_{6}{{{{{\rm{H}}}}}}_{6}}{{{{{{\mathrm{Amount}}}}}} \,{{{{{\mathrm{of}}}}}} \,{{{{{\mathrm{generated}}}}}} \,{{{{{\mathrm{phenol}}}}}} \,{{{{{\mathrm{with}}}}}}\,{{{{{\rm{C}}}}}}_{6}{{{{{\rm{D}}}}}}_{6}}$$

For example,

Over Pd_1.25_Cu_0.006_/P25:

$${{{{{\mathrm{KIE}}}}}}_{{{{{\mathrm{phenol}}}}}}=\frac{290.70}{40.21}=7.23$$

Over bare P25:

$${{{{{\mathrm{KIE}}}}}}_{{{{{\mathrm{phenol}}}}}}=\frac{131.27}{103.49}=1.27$$

### Computational details

Density-functional theory calculations were performed using CRYSTAL17 software^60^, with the PBE functional^61^ and the D3 dispersion correction^62^, with a localised basis set and pseudopotential for Pd^63^, and localised all-electron basis sets for Cu^64^, Ti and O of TiO_2_^65^, O, C and H of water and benzene adsorbates^66^ obtained from the CRYSTAL web site. TiO_2_ was modelled as a periodic slab of anatase with the (101) surface orientation and a 2 × 2 extended surface unit cell, with the thickness of 8 atomic layers, with slabs separated by a 500 Å vacuum gap in the vertical direction. Adsorbed Pd_4_ and Pd_3_Cu_1_ clusters and adsorbed molecules were placed on one side of the slab. All atoms’ positions were fully optimised. Binding energies were calculated including the basis set superposition error correction^67^.

## Supplementary information

Supplementary Information

Description of Additional Supplementary Files

Video 1

## Data Availability

The data that supports this study are available from the corresponding author upon reasonable request.
