# Supplementary material for: Highly selective oxidation of benzene to phenol with air at room temperature promoted by water
Source: Nat Commun. 2023 Jul 22;14:4431. doi: 10.1038/s41467-023-40160-w (PMC10363151; doi:10.1038/s41467-023-40160-w)
Supplement: Supplementary file 1 — Supplementary Information [file 41467_2023_40160_MOESM1_ESM.pdf]

Supplementary Materials for

Highly Selective Oxidation of Benzene to Phenol with Air at Room Temperature Promoted by Water

Jijia Xie[1], Xiyi Li[1], Jian Guo[2], Lei Luo[3], Juan J. Delgado[4,5], Natalia Martsinovich,[6] Junwang Tang[1, 7*]

Correspondence to: junwang.tang@ucl.ac.uk

This PDF file includes:

Supplementary Fig. 1 to 33
Supplementary Tables 1 to 12

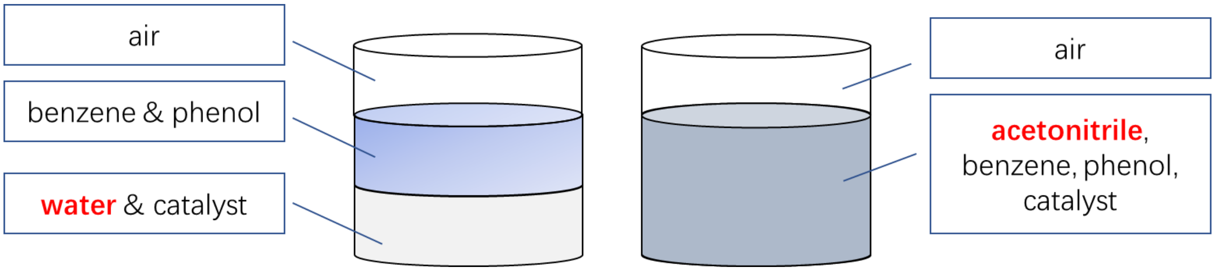

Supplementary Fig. 1. Comparison of reaction systems in the presence or absence of acetonitrile. Benzene could be readily dissolved in acetonitrile but not be dissolved in water (Left), while benzene, phenol and photocatalysts were evenly dispersed in the acetonitrile when using it as the solvent (Right). In the conventional design (Right), photocatalyst particles and the generated product phenol could not be *in-situ* separated, resulting in further oxidation of the product to CO_2. In this study, when replacing acetonitrile with water (Left), benzene and water were in a different phase and the benzene phase could fast extract phenol from the interface of benzene and water, thus avoiding the further oxidation of the product phenol.

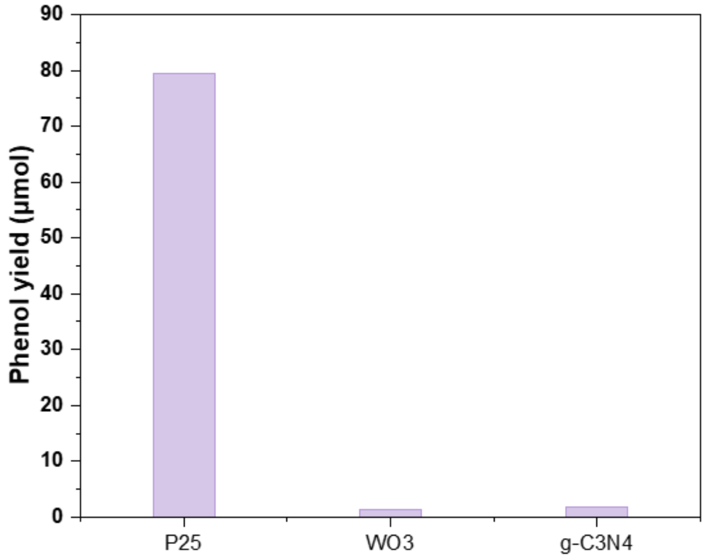

Supplementary Fig. 2. The comparison of phenol production over P25, WO_3 and g-C_3N_4. Reaction condition: 30 mg photocatalysts, 10 ml water, 20 ml benzene, pH=2 adjusted by H_3PO_4, 365 nm LED irradiation and operated at 25°C.

The production of phenol over WO_3 and g-C_3N_4 is only 1.4 and 1.9 μmol after 2 hours reaction, which is around 56 and 40 times lower than that of P25. The reason for such high activity on P25 is likely due to its intrinsic property of the relatively efficient charge separation[1]. Thus, the P25 was selected as the base for further investigation.

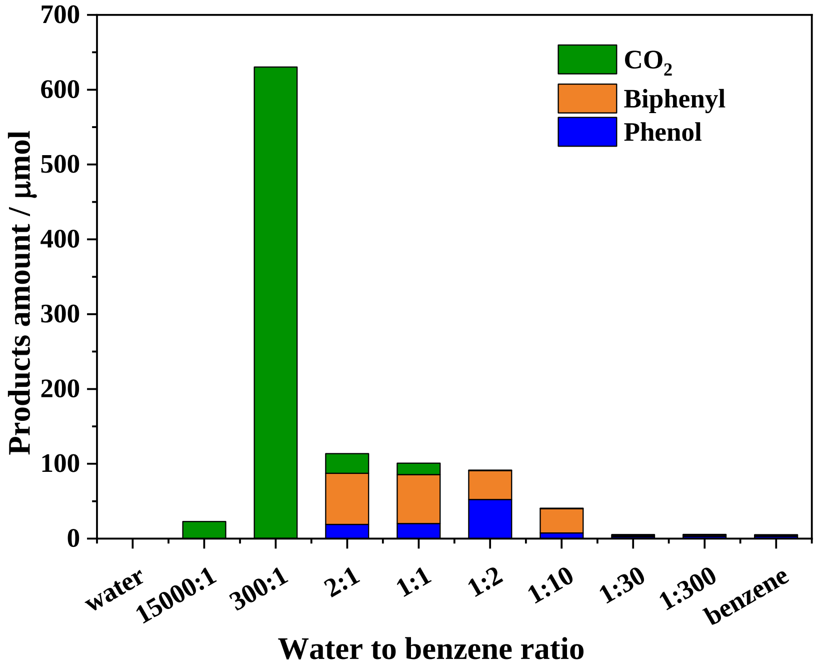

Supplementary Fig. 3 Products distribution at different water to benzene ratio over P25 photocatalyst. Reaction conditions: 30 mg P25, the overall water and benzene volume is 30 mL, pH neutral, reaction time: 2 h and 365 nm LED irradiation at 25°C.

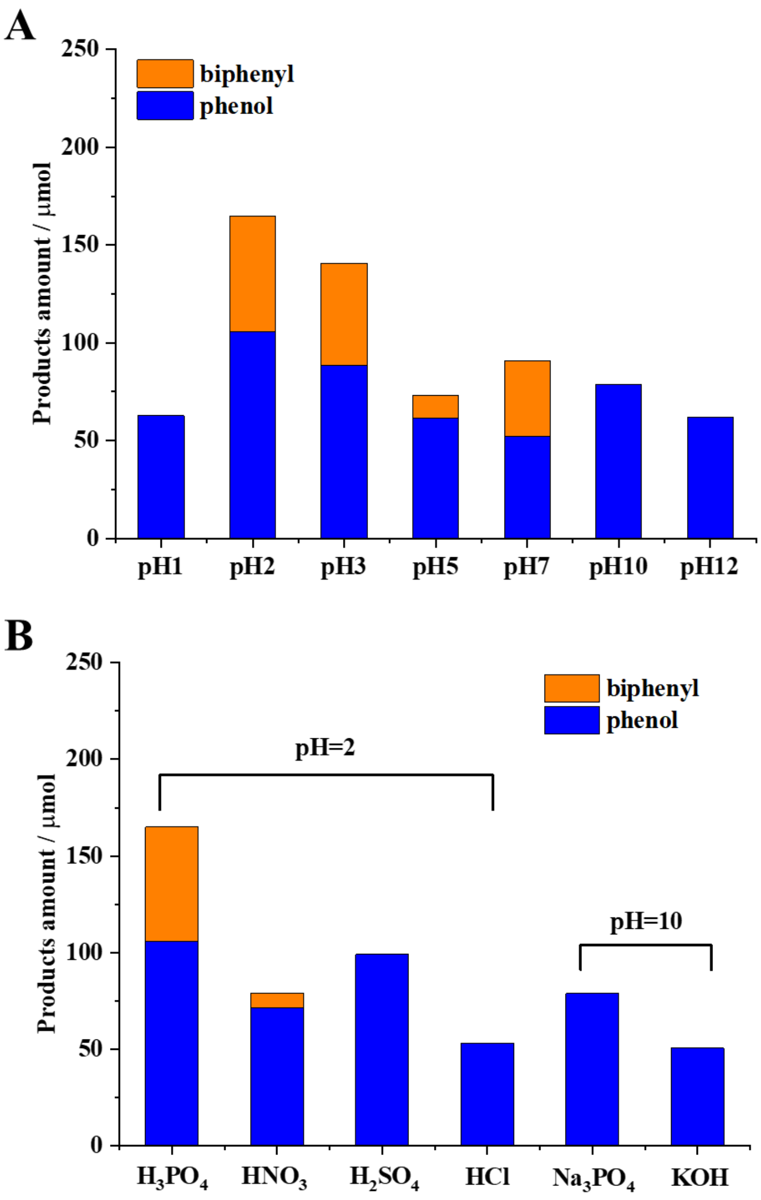

Supplementary Fig. 4 Phenol and biphenyl generation amount at different pH values over bare P25 (A) under different pH values adjusted by H_3PO4/Na_3PO_4 and (B) pH value adjusted by various reagents. Reaction conditions: 30 mg bare P25, 10 ml water, 20 ml benzene, reaction time: 2h and 365 nm LED irradiation at 25°C.

During the reaction, oxygen reduction and benzene activation promoted by water were two related half reactions. The photoelectrons would reduce oxygen to form superoxide radicals, then consuming protons to produce water and completing the catalytic cycle. Therefore, under the acidic condition, the availability of more protons could promote the consumption of superoxide radicals (the reduction half-reaction), facilitating the transfer of photoelectrons. While much more hydroxyl anions existed under alkaline conditions, this was beneficial for the generation of hydroxyl radicals to promote the oxidation half-reaction.

Supplementary Table 1. pH value before and after reaction when using different reagents over bare P25 as shown in Supplementary Fig. 2B.

Reagents	pH value before reaction	pH value after reaction
H_3PO_4	2	2
HNO_3	2	2.12
H_2SO_4	2	1.92
HCl	2	2.08
Na_3PO_4	10	7.28
KOH	10	6.54

Supplementary Table 2. Catalytic performance of various photocatalysts used in this work. Optimisation of the Pd to Cu ratio, the products selectivity is the average of three batches samples (each repeated twice) and the error bars were the standard deviation of six measurements.

Catalysts	Selectivity / %			
	phenol	biphenyl	others	CO2
Bare P25	47.41±1.42	52.04±3.35	0.15±0.02	0.41±0.08
$Pd_{0.006}Cu_{0.002}/P25$	76.48±2.29	23.10±1.54	0.17±0.03	0.24±0.05
$Pd_{0.02}Cu_{0.011}/P25$	92.69±2.78	6.37±0.99	0.33±0.05	0.61±0.12
$Pd_{1.25}Cu_{0.016}/P25$	92.30±2.45	0.00	7.30±1.17	0.40±0.08
$Pd_{2.13}Cu_{0.023}/P25$	89.26±2.68	10.05±0.82	0.44±0.07	0.25±0.05
$Pd_{3.86}Cu_{0.14}/P25$	95.45±2.86	3.62±0.11	trace	0.93±0.19

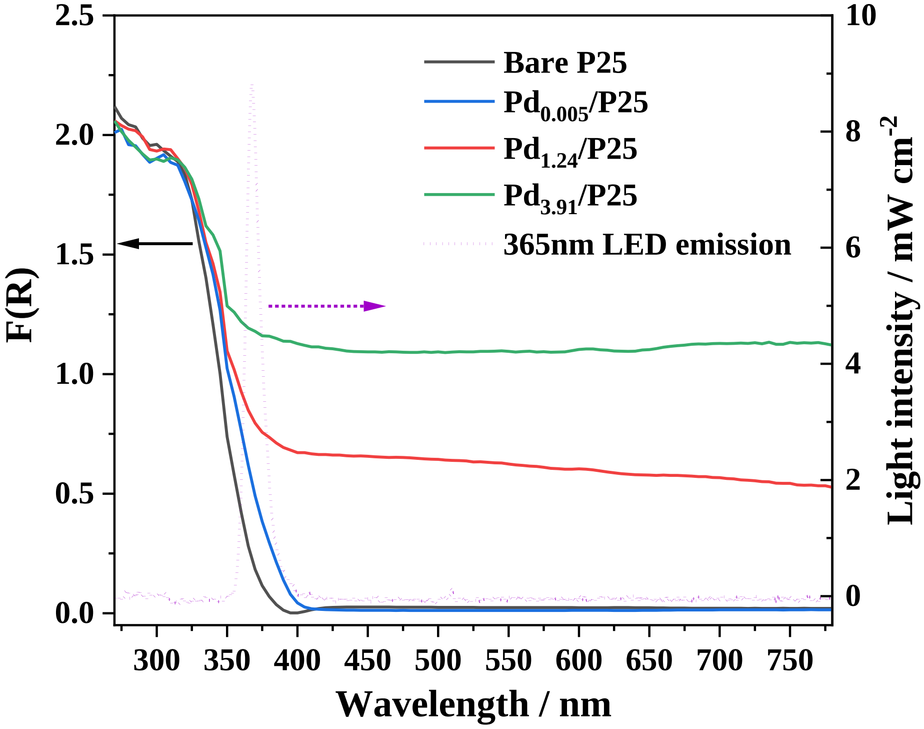

Supplementary Fig. 5 UV-Vis spectra of different Pd decorated P25 and the emission spectrum of the 365nm LED light. The background absorption (>400nm) of both Pd/P25 and Pd-Cu/P25 samples was likely induced by the Pd particles scattering

Supplementary Table 3. Summary of representative results of the phenol yields by benzene oxidation using either thermal catalysis or photocatalysis. Up to now except ours new results, none of these used the most preferable experimental conditions, including water as a solvent and O_2/air as the only oxidant operated at room temperature.

**Thermal catalysis**						
**No.**	**Catalysts**	**Oxidant**	**Solvent**	**T/°C**	**Phenol Yield mmol $(gh)^{-1}$ (selectivity)**	**Ref.**
1	Titanium silicalite	H_2O_2	sulfolane	100	2.2 (94%)	2003[2]
2	Chemically covered graphene	H_2O_2	acetonitrile	60	3.64 (99%)	2013[3]
3	Co-ISA/CNS	H_2O_2	acetonitrile	25	6.1 (88%)	2018[4]
4	Cu-SA-HCNS	H_2O_2	acetonitrile	60	5.8 (96.7%)	2018[5]
5	FeOCl	H_2O_2	acetic acid	60	12.1 (100%)	2018[6]
6	Fe-N_xC_y SAs/N-C	H_2O_2	acetonitrile	30	2.68 (100%)	2019[7]
7	Fe/ZSM-5 (2N,360)-st	N_2O	gas phase	350	7 (>99%)	2017[8]
8	Cs(1 wt %)/β	N_2O	gas phase	400	0.57 (99.9%)	2018[9]
9	Pd membrane	1:1:10 $H_2/O_2/He$	gas phase	150	15.9(85.3%)	2002[10]
10	$H_7PMo_8V_4O_{40}\cdot nH_2O$	3:2 Air/CO	acetic acid/water	90	0.68 (59%)	2005[11]
**Photocatalysis**						
**NO.**	**Photocatalysts**	**Oxidant**	**Solvent**	**Light source**	**Phenol Yield mmol/g/h (selectivity)**	**Ref.**
1	Fe-g-C_3N_4/SBA-15	H_2O_2	acetonitrile	500W Xe lamp (>420nm)	5.36 (20.7%)	2009[12]
2	Au/$Ti_{0.98}V_{0.02}O_2$	H_2O_2	acetonitrile	400W Hg lamp (200-400nm)	3.4 (88%)	2014[13]

3	Fe-CN/TS-1–2	H_2O_2	ethanol	300W Xe lamp (>420nm)	4.5 (18.4%)	2014[14]
4	MIL-100(Fe)	H_2O_2	acetonitrile	300W Xe lamp (420-800nm)	1.28 (96%)	2015[15]
5	Au-Pd/g-C_3N_4	H_2O_2	acetonitrile	100 W Hg lamp with a cut off filter	134.8(98%)	2018[16]
6	CuPd/C_3N_4	H_2O_2	acetonitrile	Solar simulator	179 (89.6%)	2019[17]
7	TiO_2@MCF/CH_3/UV (23.3)	Air	acetonitrile	300W Xe lamp (>320nm)	6.25×10^{-3} (49.7%)	2011[18]
8	$[RuII(Me_2phen)^3]^{2+}$ & $[CoIII(Cp^*)(bpy)(H_2O)]^{2+}$	O_2	Acetonitrile (homogen-erous system)	Xe lamp (Full Arc)	0.02×10^{-3} (unreported)	2017[19]
9	Bi_2WO_6/$CdWO_4$	O_2	acetonitrile	300W Xe lamp (>400nm)	0.19 (>99%)	2018[20]
10	$Pd_{1.25}Cu_{0.016}$/P25	Air	water	5W LED (365nm)	4.8 (93%)	This work

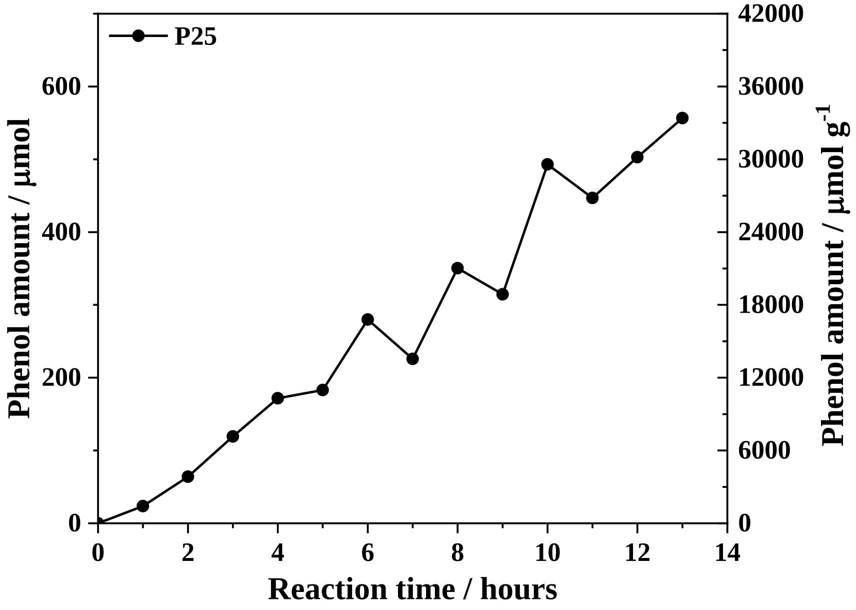

Supplementary Fig. 6 Phenol generation rate over bare P25. Reaction conditions: 30 mg photocatalysts, 10 ml water, 20 ml benzene, pH=2 adjusted by H_3PO_4, 365 nm LED irradiation and operated at 25ºC.

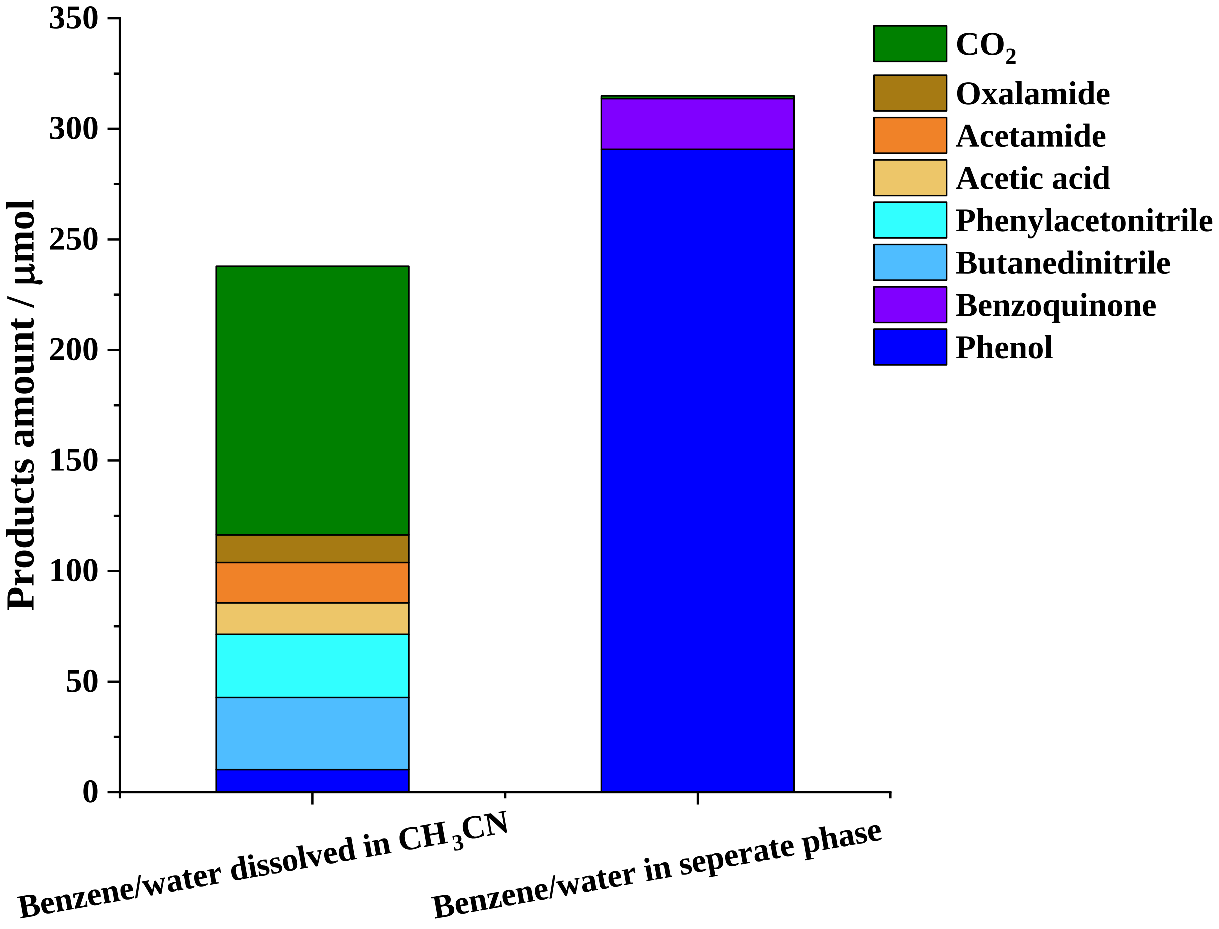

Supplementary Fig. 7. Products generation amount in homogeneous and heterogeneous reactant-solvent system over $Pd_{1.25}Cu_{0.016}$/P25 Left column: The solvent was mixed by 10ml CH_3CN, 20ml benzene and 10ml water to form a homogeneous reactant-solvent system. Right column: 20ml benzene and 10ml water were in two phases. Reaction conditions: 30 mg $Pd_{1.25}Cu_{0.016}$/P25, reaction time: 2h, 365 nm LED irradiation and operated at 25°C.

A: Butanedinitrile

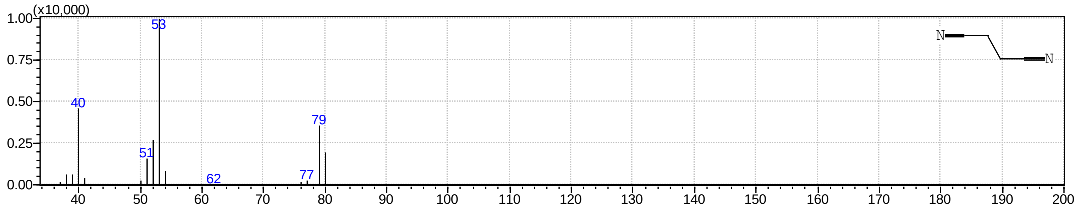

B: Phenylacetonitrile

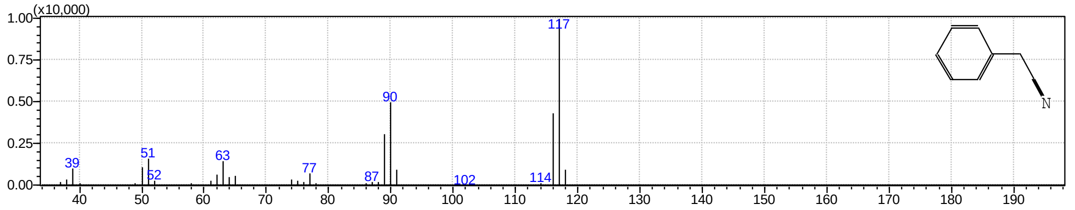

C: Acetamide

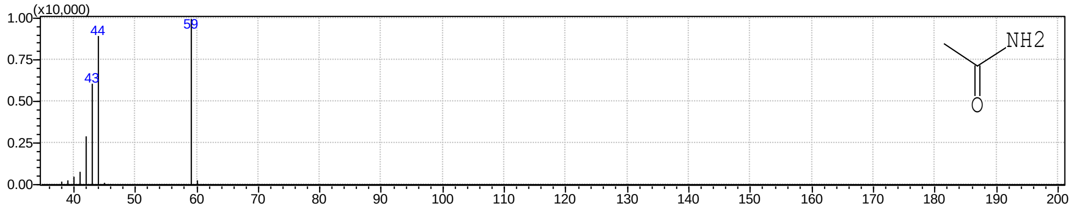

D: Oxalamide

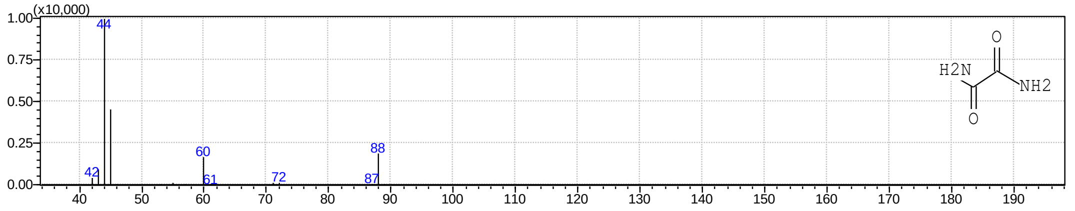

Supplementary Fig. 8 Mass spectra of the additional products ((A) Butanedinitrile, (B) Phenylacetonitrile, (C) Acetamide and (D) Oxalamide) when using acetonitrile as extra solvent as shown in Supplementary Fig. 7.

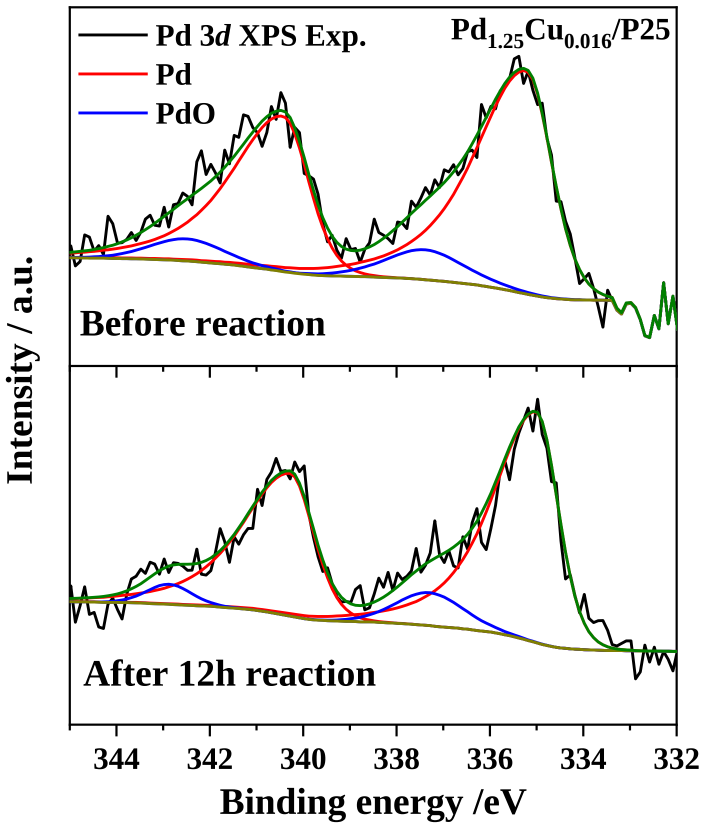

Supplementary Fig. 9 Pd 3*d* XPS of $Pd_{1.25}Cu_{0.016}$/P25 before (top) and after 12h reaction (bottom).

Supplementary Table 4 Pd 3$d_{5/2}$ XPS of $Pd_{1.25}Cu_{0.016}$/P25 before (bottom) and after 12h reaction (top).

	Peak	Position/eV	FWHM/eV	Percentage
Pre-reaction	Pd(0)	335.39	1.42	56.4%
	Pd(II)	336.86	2.35	43.6%
Post-reaction	Pd(0)	335.27	1.42	56.8%
	Pd(II)	337.16	2.35	43.2%

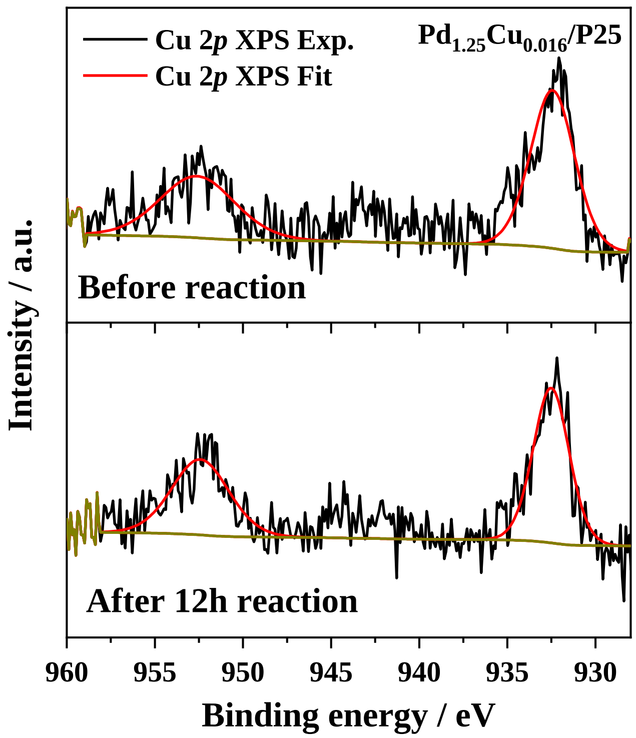

Supplementary Fig. 10. Cu 2*p* XPS of $Pd_{1.25}Cu_{0.016}$/P25 before (top) and after 12h reaction (bottom).

Supplementary Table 5 Cu $2p_{3/2}$ XPS of $Pd_{1.25}Cu_{0.016}$/P25 before (bottom) and after 12h reaction (top).

	Peak	Position/eV	FWHM/eV	Percentage
	Cu(0)	931.80	1.79	58.6%
Pre-reaction	Cu(II)	933.76	2.93	41.4%
	satellite	943.20	/	/
	Cu(0)	932.0	1.79	58.4%
Post-reaction	Cu(II)	933.59	2.93	41.6%
	satellite	943.20	/	/

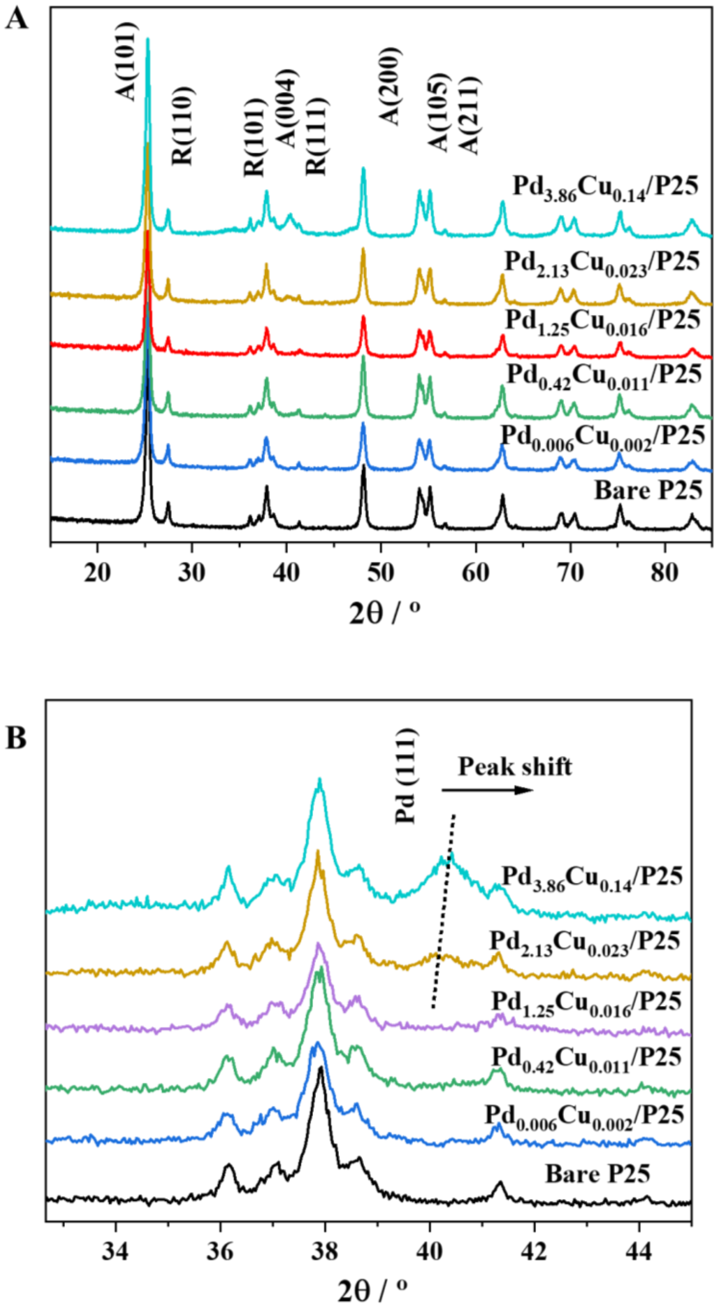

Supplementary Fig. 11 (A) PXDR patterns and (B) zoomed PXRD of different catalysts. All samples presented the typical P25 diffraction indicating a stable structure of the P25 framework during the cocatalysis decoration (anatase: JCPDS card no. 21-1272; Rutile: JCPDS card no. 21-1276). When increasing the Pd ratio to 2.13 wt.%, an additional peak located at *ca*. $2\theta=40^{o}$ was observed which could be assigned as Pd (111) of metallic palladium (JCPDS 87-0638). When introducing more cocatalysts, the peak shifted to a higher degree indicating a stronger interaction between cooper and palladium as reported previously[17,21].

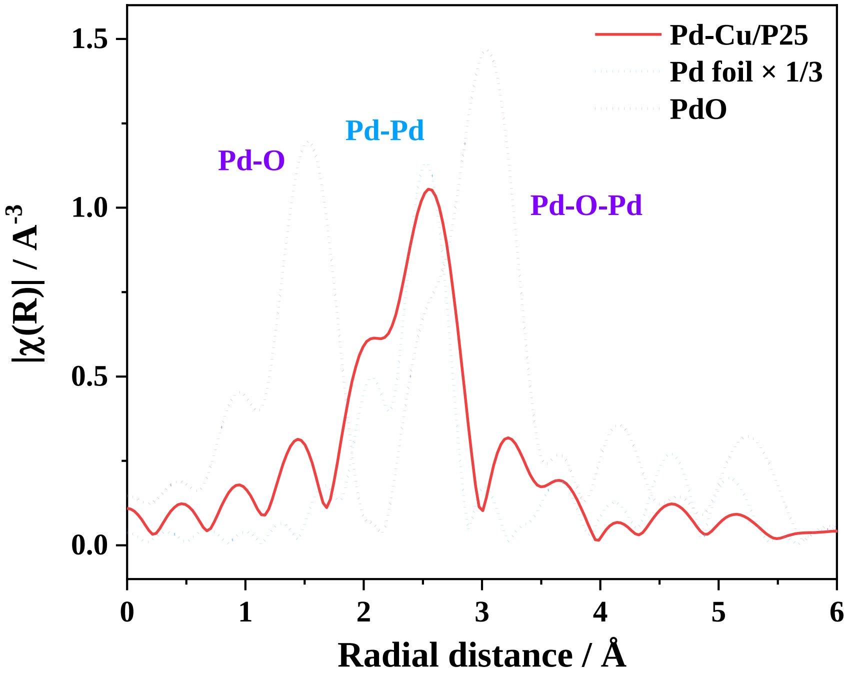

Supplementary Fig. 12. Pd K-edge EXAFS spectra of $Pd_{1.25}Cu_{0.016}$/P25, Pd foil and PdO standard sample.

Supplementary Table 6 Fitting results of palladium K-edge EXAFS spectra of $Pd_{1.25}Cu_{0.016}$/P25.

Shell	Bond length/Å	Coordination number	σ^2 (Å^2)	R-factor
Pd-Pd	2.73±0.01	5.7±0.01	0.007±0.001	
Pd-Cu	2.63±0.03	0.95±0.01	0.008±0.005	0.009
Pd-O	1.99±0.05	0.95±0.01	0.010±0.007	

σ^2 represents the mean squared displacement.

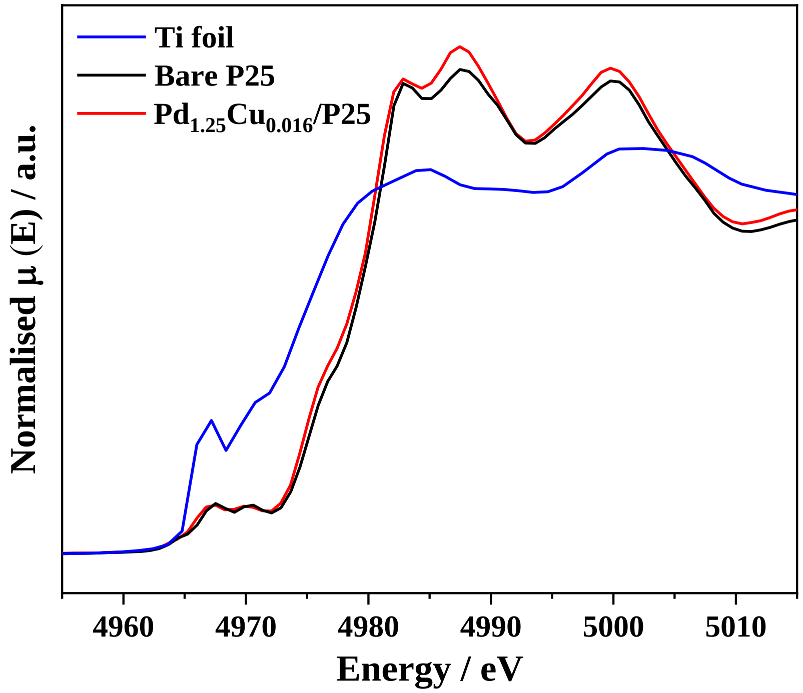

Supplementary Fig. 13 Ti K-edge XANES spectra of Ti foil, P25 and $Pd_{1.25}Cu_{0.016}/P25$

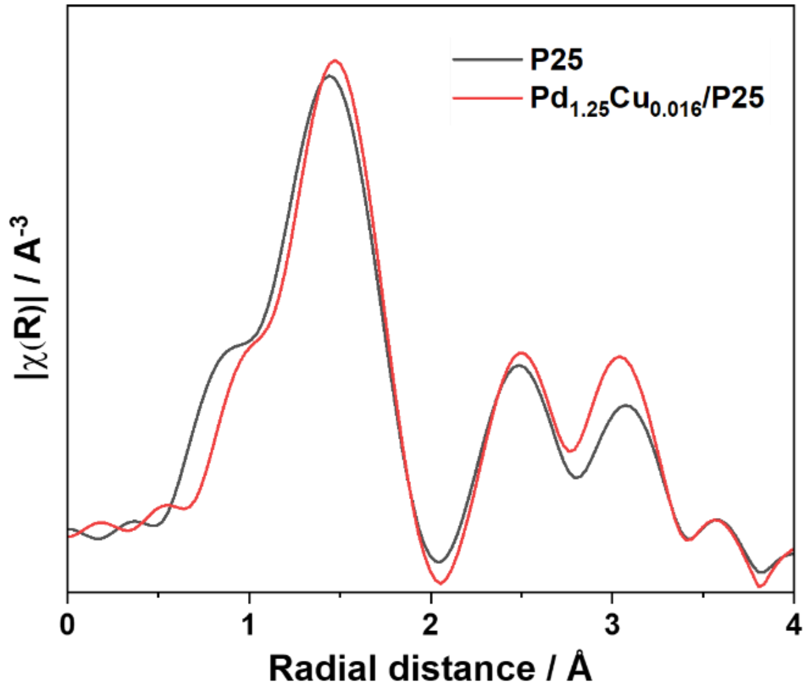

Supplementary Fig. 14. Ti K-edge EXAFS spectra of P25 and $Pd_{1.25}Cu_{0.016}$/P25

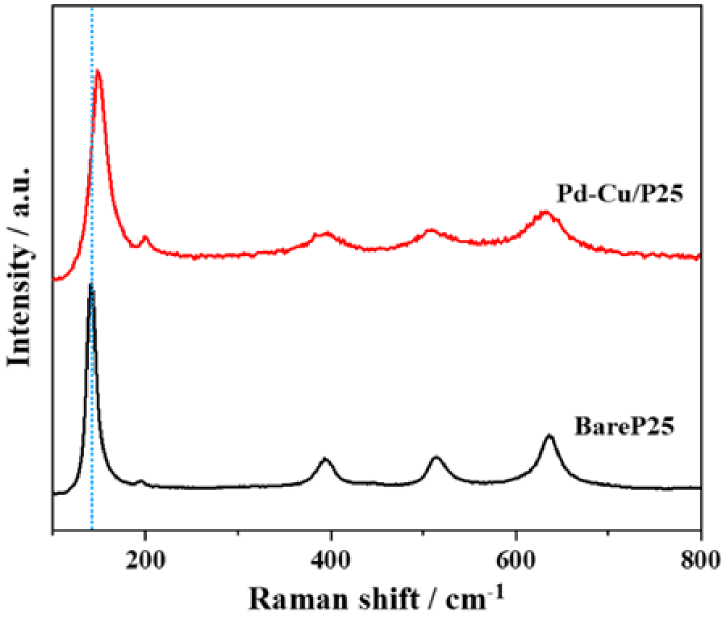

Supplementary Fig. 15 Raman spectra of PdCu-P25 and bare P25. An obvious blueshift of the E_g peak located at ca. 144 cm^{-1} was observed. This peak was mainly attributed to the symmetric stretching vibration of O-Ti-O in TiO_2 [23]. The blue shift could be explained by two possible reasons: a phonon confinement effect and the strain by surface coating. Considering the photodeposition process of PdCu would not change the particle dimension of the TiO_2 itself, the photon confinement by the decrease to nanometer scale could be excluded. In contrast, the coating of PdCu nanoparticles would produce a compressive stress on the first several layer atoms of TiO_2[24]. Thus, the surface atoms of TiO_2 packed closely, leading to higher vibrational wavenumbers (blueshift)[25]. This result indicated the decoration of PdCu nanoparticles on TiO_2.

A

B

C

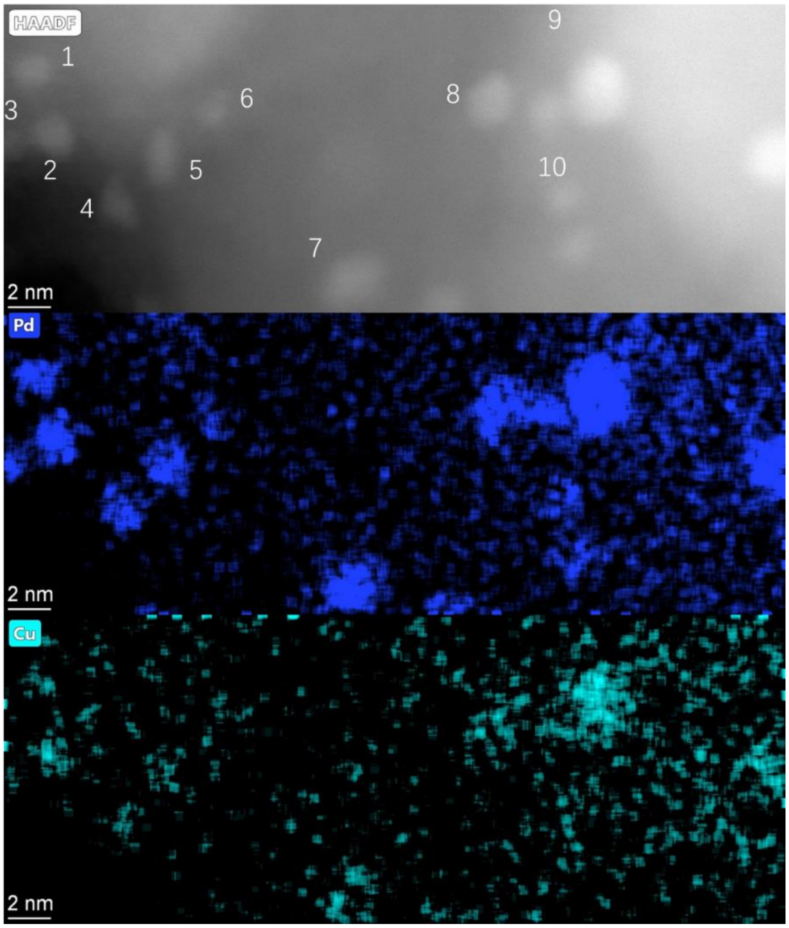

Supplementary Fig. 16 (A) HAADF-STEM images; (B,C) EDX element mapping of $Pd_{1.25}Cu_{0.016}$/P25. The sample contains 2-6 nm Pd-Cu bimetallic particles.

Supplementary Table 7. Pd to Cu ratio of $Pd_{1.25}Cu_{0.016}$/P25 detected by EDX as shown in Fig. S5. (Note: the sample contains Pd 1.25 wt% and Cu 0.016 wt%, equals to an atomic ratio of 1:0.02)

Particle	% atomic Pd	% atomic Cu
1	60	40
2	55	45
3	58	42
4	50	50
5	50	50
6	65	35
7	60	40
8	65	35
9	60	40
10	80	20

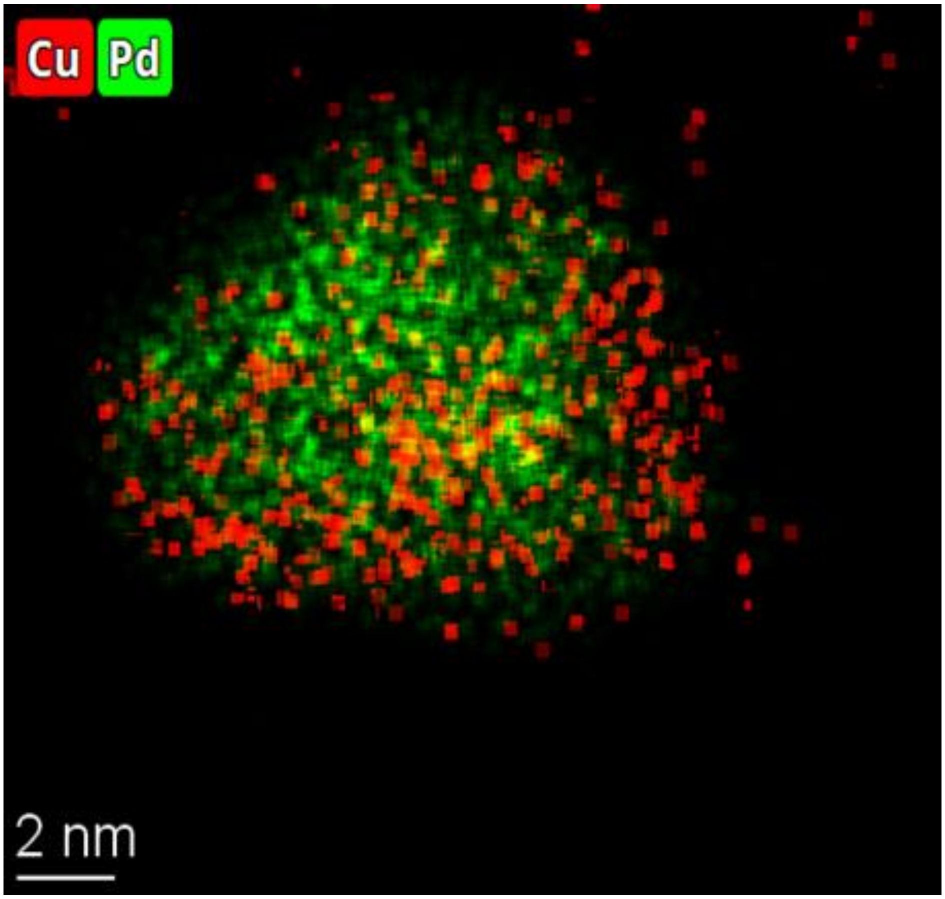

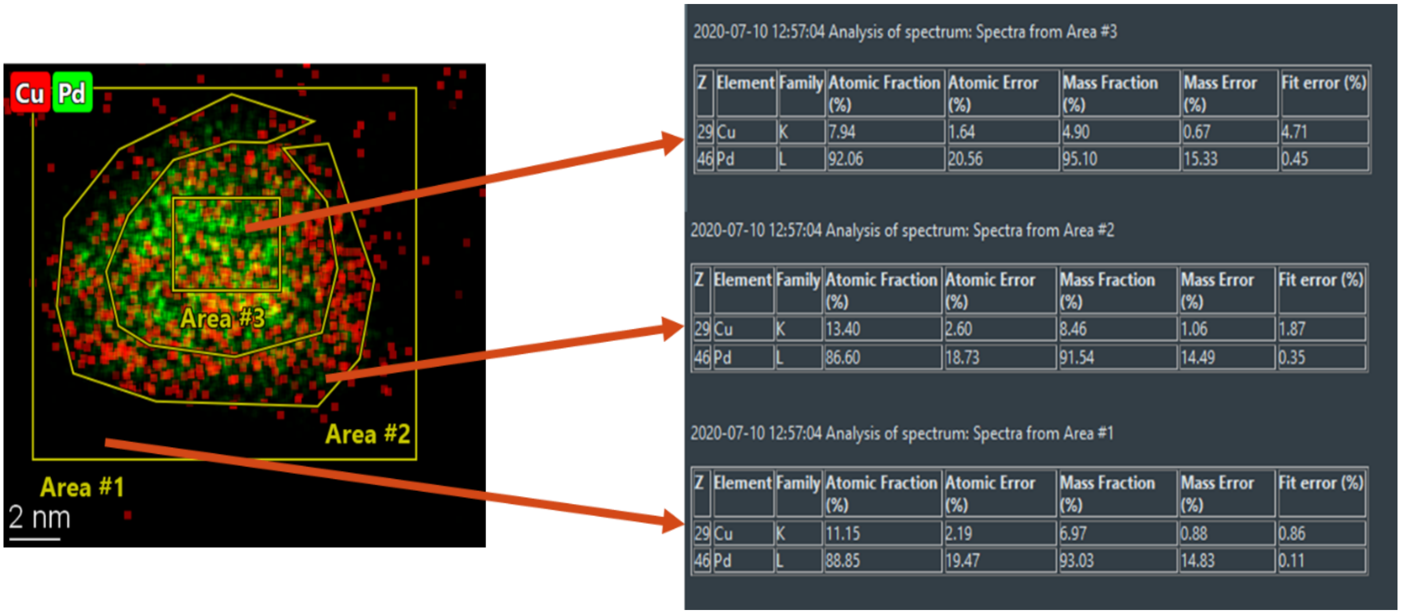

2020-07-10 12:57:04 Analysis of spectrum: Spectra from Area #3

Z	Element	Family	Atomic Fraction (%)	Atomic Error (%)	Mass Fraction (%)	Mass Error (%)	Fit error (%)
29	Cu	K	7.94	1.64	4.90	0.67	4.71
46	Pd	L	92.06	20.56	95.10	15.33	0.45

2020-07-10 12:57:04 Analysis of spectrum: Spectra from Area #2

Z	Element	Family	Atomic Fraction (%)	Atomic Error (%)	Mass Fraction (%)	Mass Error (%)	Fit error (%)
29	Cu	K	13.40	2.60	8.46	1.06	1.87
46	Pd	L	86.60	18.73	91.54	14.49	0.35

2020-07-10 12:57:04 Analysis of spectrum: Spectra from Area #1

Z	Element	Family	Atomic Fraction (%)	Atomic Error (%)	Mass Fraction (%)	Mass Error (%)	Fit error (%)
29	Cu	K	11.15	2.19	6.97	0.88	0.86
46	Pd	L	88.85	19.47	93.03	14.83	0.11

Supplementary Fig. 17. EDX element mapping and Cu to Pd ratio of a selected Cu-Pd bimetallic particle shown in Fig. 2D.

Supplementary Table 8 The detected Pd to Cu ratio of $Pd_{1.25}Cu_{0.016}/P25$ by different technologies.

Technology	ICP-AES	EDX	XPS
Agreed detection depth	Bulk	1-3 μm	<5 nm
Cu : Pd atomic ratio	0.02 : 1	> 0.25 : 1	1.25 : 1

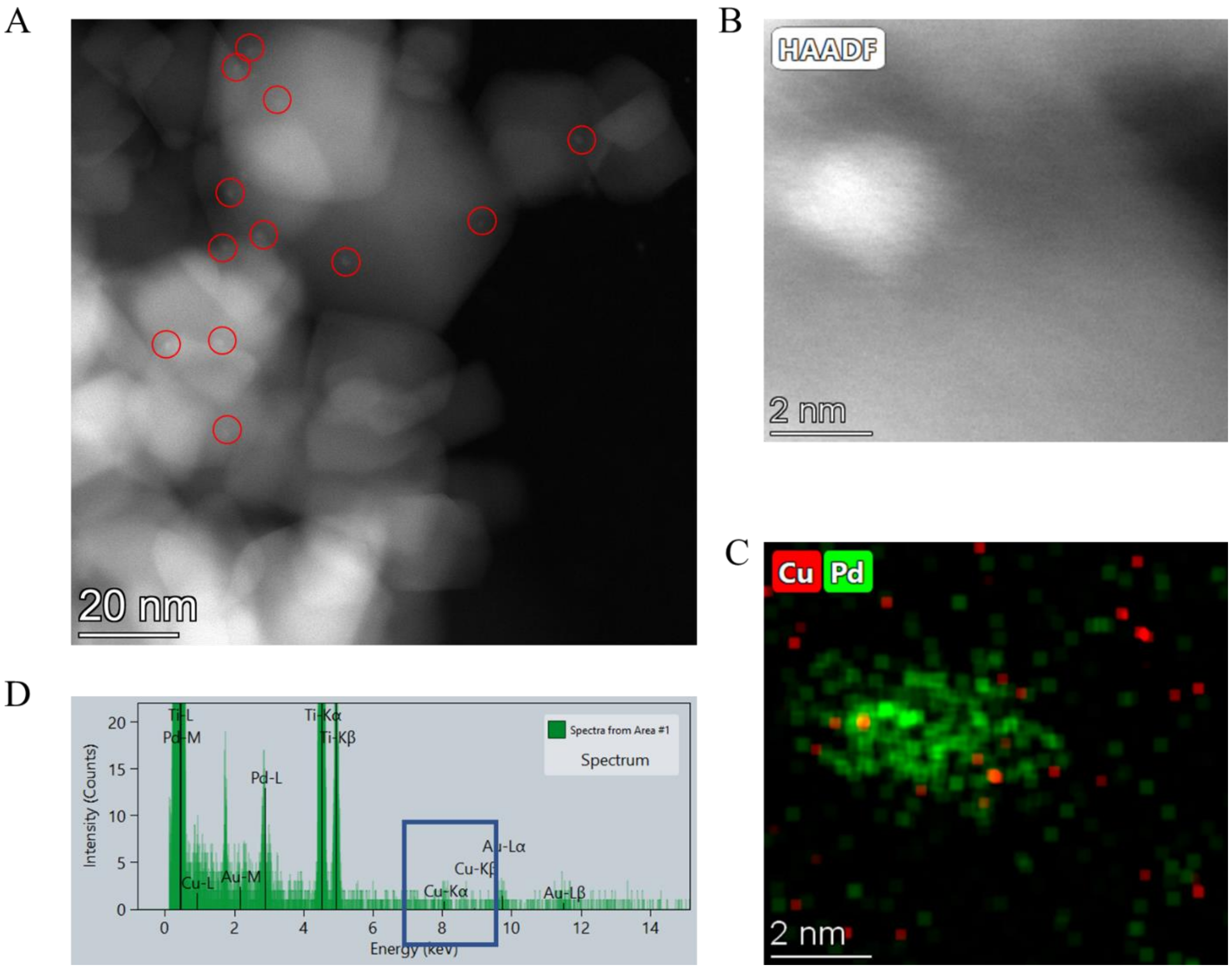

Supplementary Fig. 18 (A) HAADF-STEM images; (B) zoomed HAADF-STEM images; (C) EDX element mapping of the zoomed image and (D) area selected EDX spectrum of $Pd_{0.006}Cu_{0.002}$/P25. Such results suggested that Cu signal was detectable, but the quantification was impossible on such low loading amount of 0.002 wt%.

Supplementary Table 9 O18 to O16 labelled phenol ratio in the product when benzene reacted with O18 labelled water over Pd1.25Cu0.016/P25.

Time	O18 to O16 labelled phenol ratio
0 h	0
0.5 h	5.38
1 h	5.59
1.5 h	5.84
2 h	5.96

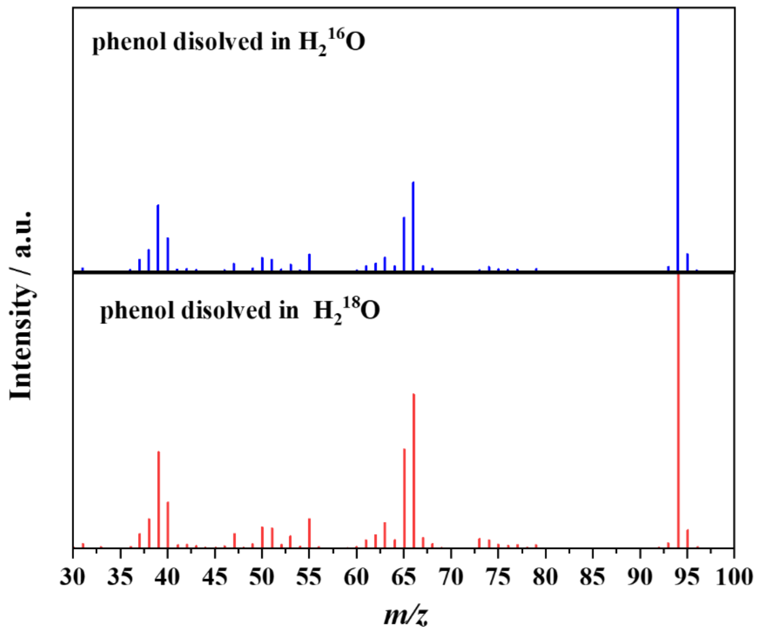

Supplementary Fig. 19. The comparison of phenol mass spectra: Phenol dissolved in $H_2{}^{16}O$ (top panel) and $H_2{}^{18}O$ (bottom panel) after 2 hours.

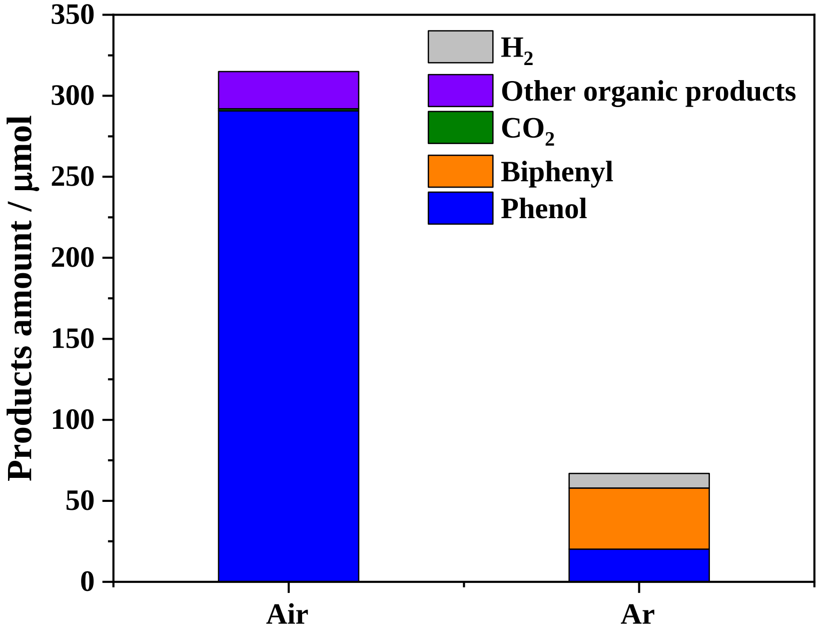

Supplementary Fig. 20 Photocatalytic benzene conversion in air and inert Ar atmosphere over $Pd_{1.25}Cu_{0.016}$/P25.

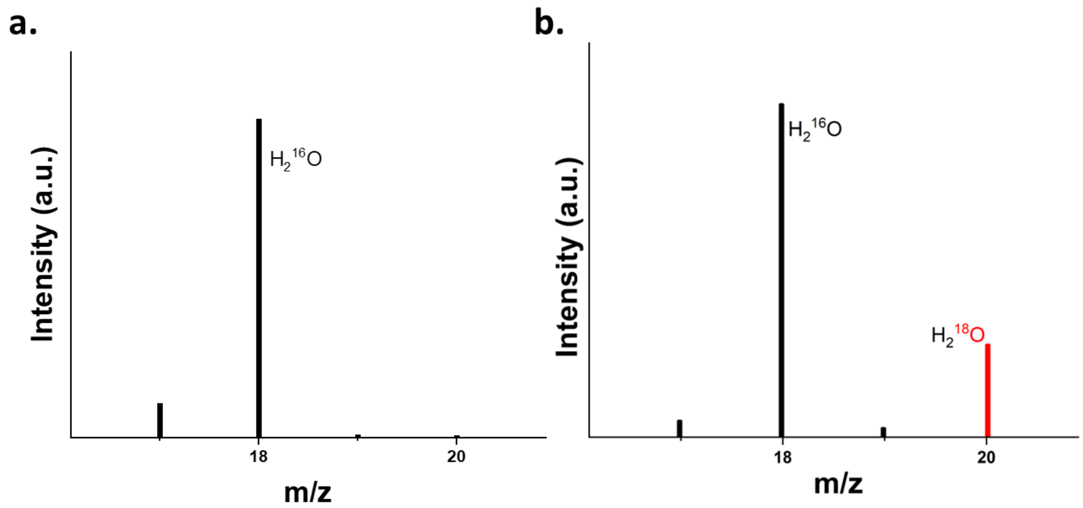

Supplementary Fig. 21 Blank mass spectra (a) or mass spectra of products using $^{18}O_2$ as feed gas (b).

Considering the stoichiometric reaction, the generation of water should be around 150 μmol for the optimum PdCu/P25 catalyst. This is more than thousand times lower than that of the reaction solvent (10 mL water/555, 555 μmol). We tried to use the $^{18}O_2$ isotope labelling gas to perform the reaction, but our mass spectrometry is not sensitive enough to distinguish such small amount of $H_2{}^{18}O$ in nearly 10ml of $H_2{}^{16}O$. Thus, it is difficult to directly identify the generation of water in our reaction system. However, the reduction of O_2 to H_2O can be proved via indirect experimental evidence in our lab. Methane with stronger C-H bond than benzene was used as the reactant (hole scavenger) to avoid the involvement of large amount of water in this case. The $^{18}O_2$ as feedstock gas and similar catalyst PdCu/TiO_2 were introduced to run a gas-solid phase reaction. The gas phase product was analysed by the mass spectrometry, as shown in Supplementary Fig. 21. It should be noted that the background of mass spectra can always have the signal for m/z = 18 due to the inevitable tiny leak from atmosphere no matter how much vacuum was applied (Supplementary Fig. 21a). Expectedly, the m/z of 18 assigned to $H_2{}^{16}O$ shows similar intensity in both background mass spectra and the mass spectra when using $^{18}O_2$ as a feedstock. Furthermore, the new signal (m/z = 20) corresponding to $H_2{}^{18}O$ appears, solidly indicates the formation of water during the reaction process. Therefore, we proposed that the photoinduced electron can reduce O_2 to form H_2O.

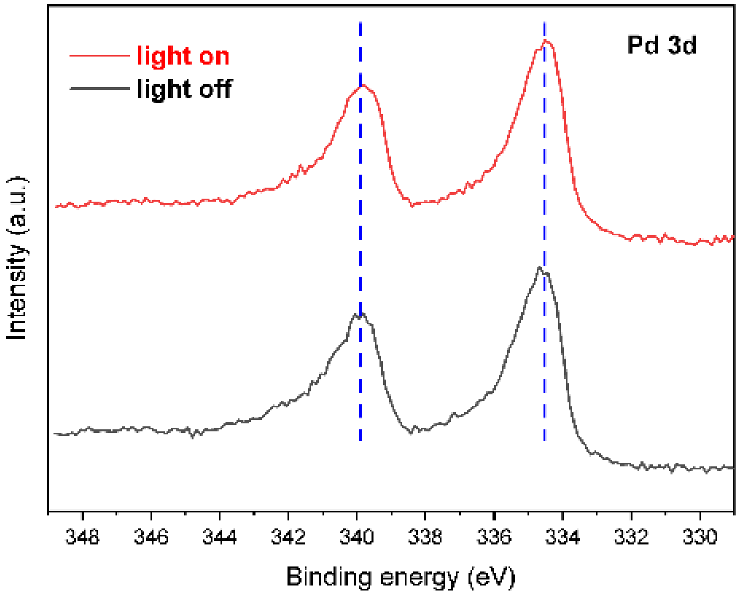

Supplementary Fig. 22. In situ Pd 3*d* XPS spectra of $Pd_{1.25}Cu_{0.016}$/P25 under light and dark conditions.

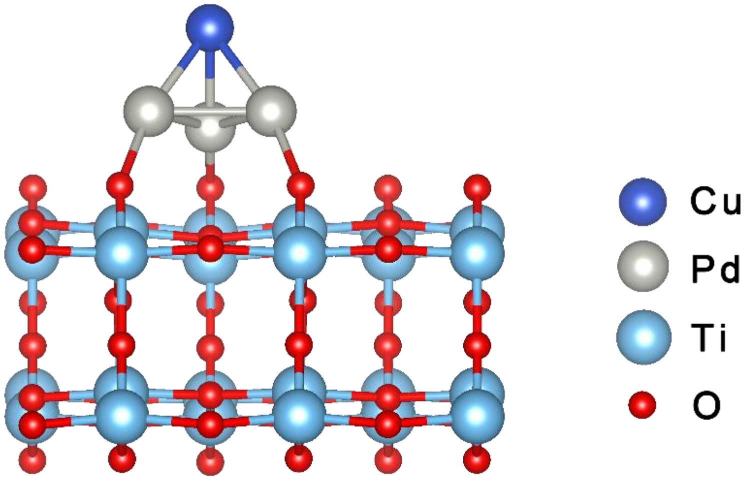

Supplementary Fig. 23. Structure of Cu-Pd cluster on TiO_2 surface for DFT calculation.

Supplementary Table 10. Charge transfer from adsorbed metal atoms to TiO_2

Adsorbate	Atom	Δq	Δq(Sum)
Pd_3Cu tetrahedron	Pd	-0.138	-0.346 (Sum of Pd)
	Pd	-0.133	
	Pd	-0.075	
	Cu	0.436	0.09 (Sum of Pd_3Cu)

*$\Delta q = q_{element} - q_{adsorbed\ atom}$

where $q_{element}$ is the formal nuclear charge of the Cu or Pd atom and $q_{adsorbed\ atom}$ is the calculated Mulliken charge of the adsorbed Cu or Pd atoms. The negative Δq indicates gain of electron density compared to an isolated atom, while the positive Δq indicates loss of electron density.

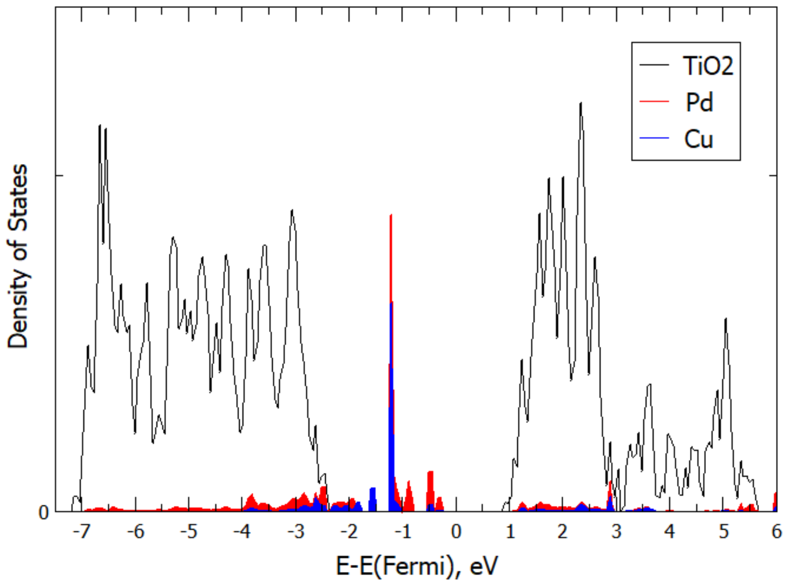

Supplementary Fig. 24. Calculated density of electronic states for TiO_2 with an adsorbed Pd_3Cu cluster. The structure of the cluster was shown in Supplementary Fig. 23. Shaded red and blue areas were the contributions of the Pd and Cu atoms, respectively. The black line showed the states contributed by TiO_2. The zero energy was at the Fermi level.

Supplementary Table 11 Binding energy (eV) of water on Pd_4 cluster and Pd_3Cu_1 cluster.

Support:	Binding energy of water (eV)
Pd_4 cluster	-0.46
Pd_3Cu_1 cluster	-0.65

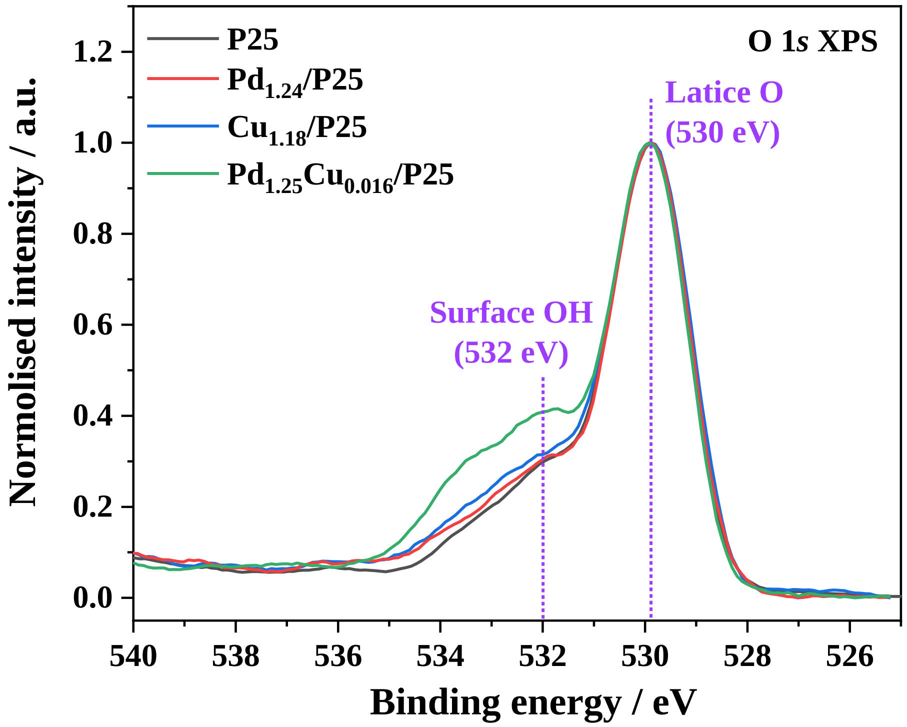

Supplementary Fig. 25 O 1*s* XPS spectra of various photocatalysts.

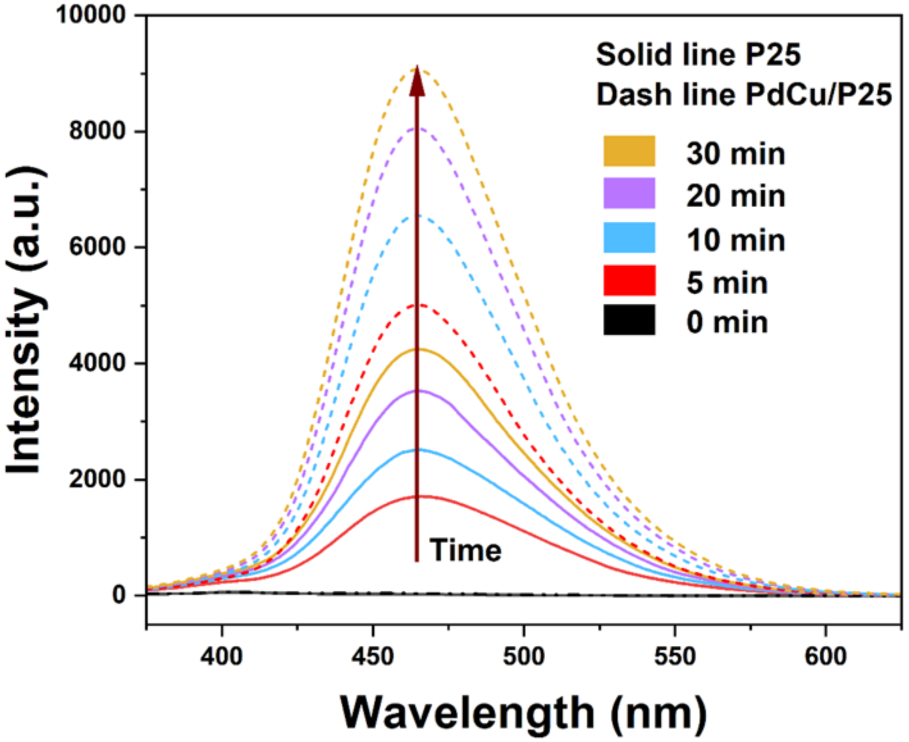

Supplementary Fig. 26 Fluorescence spectra of 7-hydroxycoumarin obtained after 300 W Xenon light irradiation in the P25 suspension and the PdCu/25 suspension (20 mg catalyst + 100 mL 0.5 mM coumarin aqueous solution). The excitation wavelength was set to 350 nm.

A study on the production of ·OH radicals was carried out on the optimised photocatalyst as it was the first intermediate of water oxidation by photoholes. The experiment was first carried out over P25 by ·OH capturing with coumarin, as shown in Supplementary Fig. 26. The ·OH radicals were detected using the coumarin fluorescence probe techniques, since coumarin reacted with ·OH radicals to form 7-hydroxycoumarin that emitted the strong fluorescence at the wavelength around 450 nm [26]. At time zero, there is no 7-hydroxycoumarin absorption peak and it becomes stronger and stronger with irradiation time, consistent with the published to prove ·OH radicals production under light irradiation [27–29]. Then the similar experiments were carried out on PdCu/P25. The intensity of fluorescence keeps increasing with time and more importantly PdCu/P25 represents a much stronger intensity of 7-hydroxycoumarin absorption than P25. The intensity achieved on PdCu/P25 after 5 minutes irradiation is even higher than that of P25 suspension after 30 minutes, proving a much higher yield rate of ·OH radicals after the decoration of PdCu on TiO_2 due to efficient holes transfer to PdCu for water oxidation to the intermediates.

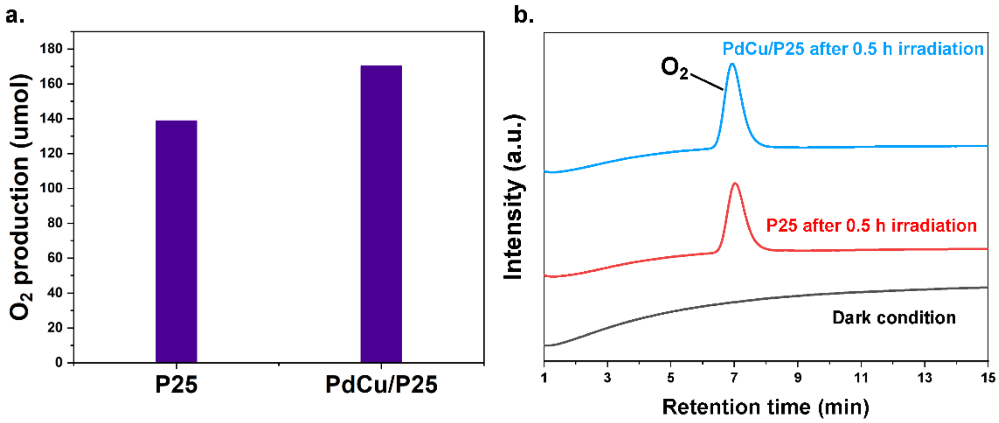

Supplementary Fig. 27 Oxygen production over P25 and PdCu/P25 after 30 minutes light irradiation (a) and their related GC spectra with the reference spectrum under dark condition (b). (Reaction condition: 100 mg catalyst, 100 mL H_2O, 100 mM $AgNO_3$ and 300 W Xenon lamp)

The experiment of water oxidation to the final product oxygen gas was also conducted, as shown in Supplementary Fig. 27. It was understandable that O_2 could be produced by P25 photocatalyst in the presence of an electron scavenger $Ag+$ ions[30]. Notably, PdCu/P25 show much higher production of O_2 compared with TiO_2 itself. This, together with the above Fig. 26, indicates that O_2 gas production is more difficult than ·OH radical production as the former requires 4-hole chemistry.

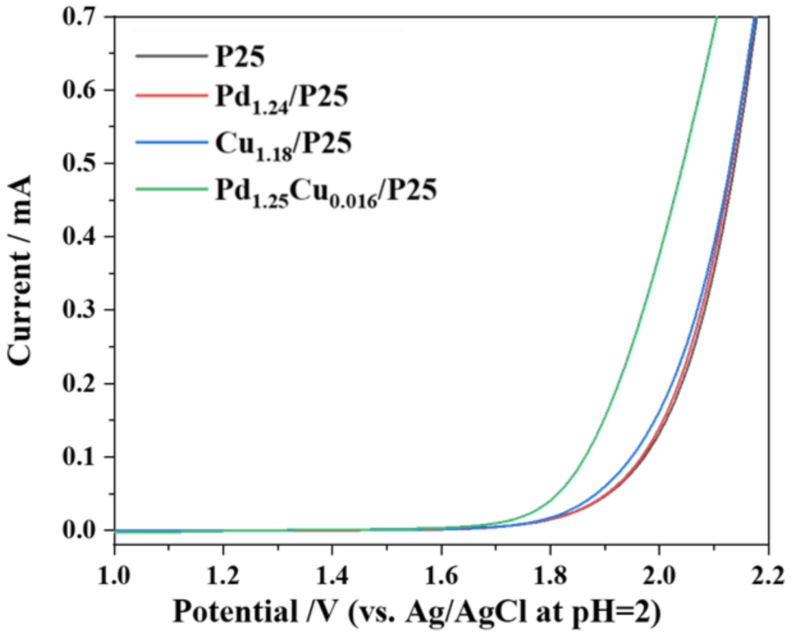

Supplementary Fig. 28 Electrochemical water oxidation spectra over various photocatalysts in H_3PO_4 aqueous solution at pH=2.

Supplementary Fig. 28 shows the water oxidation potentials over the bare P25 and decorated P25 samples. The onset potential shifts slightly negatively only when Cu species is added, which is consistent with the role of Cu as a hole acceptor and an active site to promote the water oxidation mentioned above. Moreover, the formation of PdCu structure can further shift the onset potential more negatively than that of Cu single metal, suggesting a synergy effect between two species. However, it should be careful that the copper around zero valence might be oxidised instead of water oxidation, leading to the negatively shift of onset potential. In order to confirm the copper valance during light-driven reactions, a long-time photocatalytic reaction was carried out, and the Cu species were analysed by XPS before and after the experiment oxidation process, as shown in Supplementary Fig. 10. No shift of the Cu 2p peak could be observed after the photocatalytic oxidation process, indicating the high stability of the PdCu clusters.

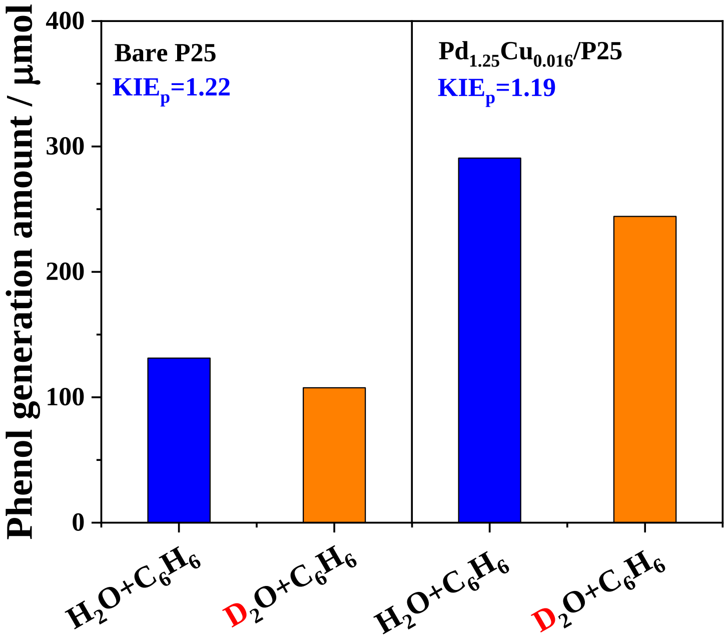

Supplementary Fig. 29. Kinetic isotopic effects of phenol generation using deuterated water as reactants over $Pd_{1.25}Cu_{0.016}$/P25 and bare P25 at pH=2.

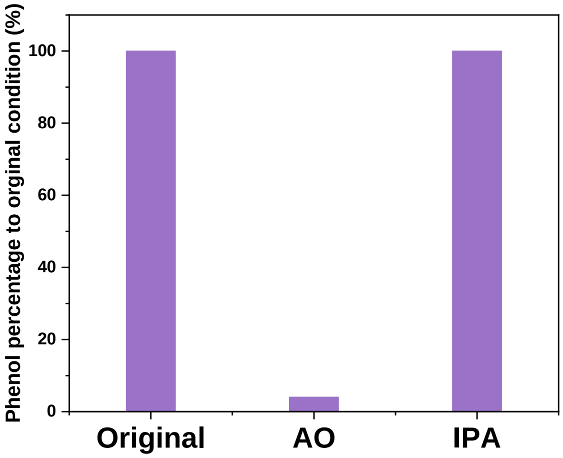

Supplementary Fig. 30 The comparison of phenol production percentage with the addition of 10 mM hole scavenger (AO) and hydroxyl radical scavenger (IPA). (Reaction condition: 30 mg photocatalysts, 10 ml water, 20 ml benzene, pH=2 adjusted by H_3PO_4, 365 nm LED irradiation and operated at 25°C)

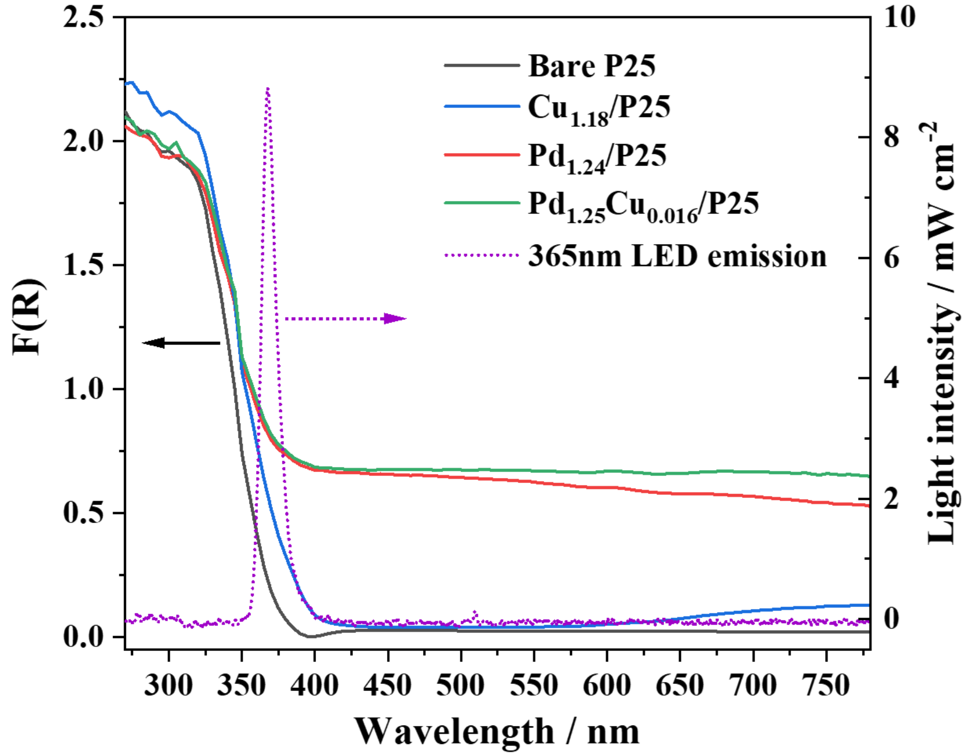

Supplementary Fig. 31 UV-Vis spectra of cocatalysts decorated P25 and the emission spectrum of the 365nm LED light. The background absorption (>400nm) of both Pd/P25 and Pd-Cu/P25 samples was likely induced by the Pd particles scattering

Pd-Cu/SiO_2 was synthesised with a similar loading ratio to $Pd_{1.24}Cu_{0.016}$/P25 using a $NaBH_4$ reduction method. After a 2-hour reaction, only a trace amount (approximately 0.4 µmol) of phenol were produced. The great difference (a factor of 700) of the phenol generation between Pd-Cu/SiO_2 (0.4 µmol) and $Pd_{1.24}Cu_{0.016}$/P25 (290 µmol) suggested that the contribution from the d-band excitation of Pd-Cu was negligible in the current reaction.

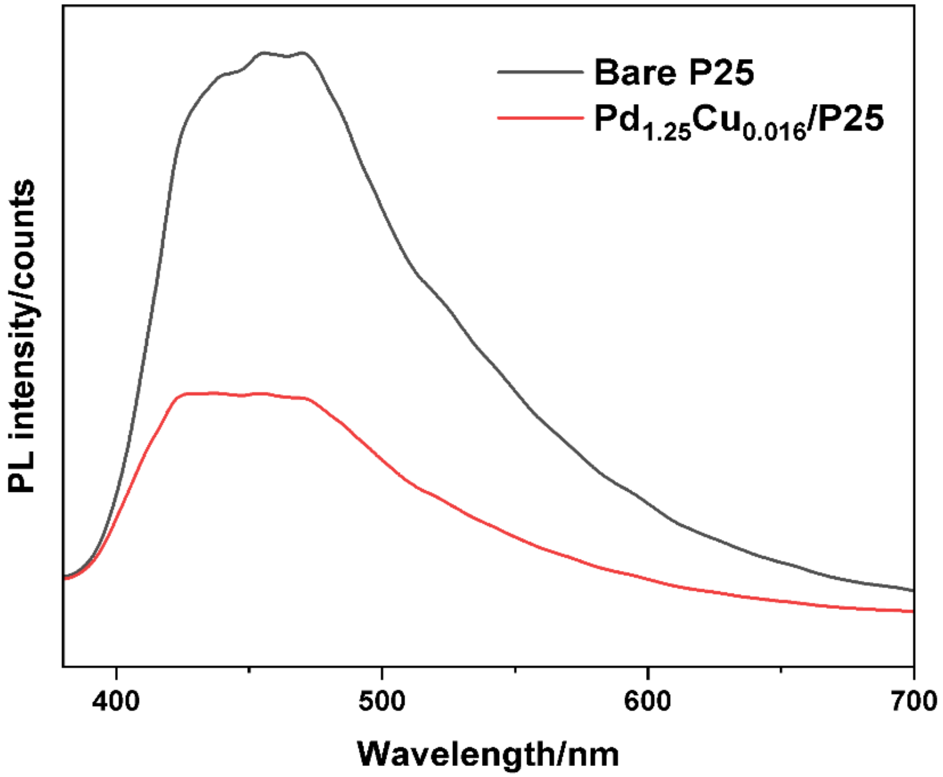

Supplementary Fig. 32 Photoluminescence spectra of bare P25 and $Pd_{1.25}Cu_{0.016}$/P25, excited by 365 nm laser.

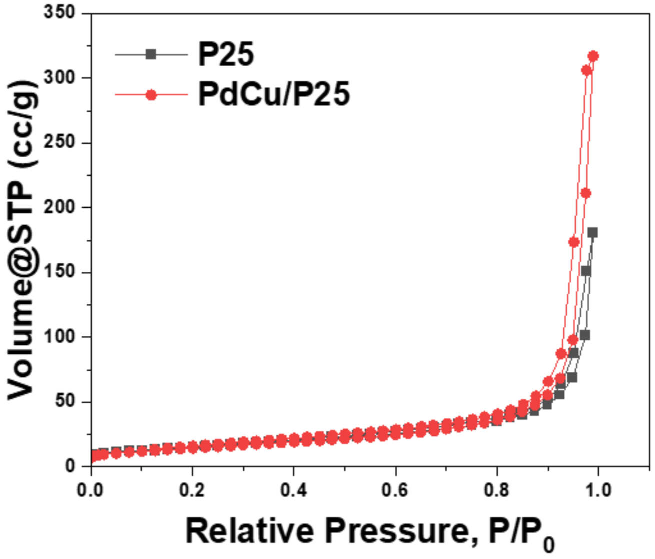

Supplementary Fig. 33 Nitrogen adsorption-desorption isotherms of P25 and PdCu/P25.

Supplementary Table 12 BET surface area and pore volume of P25 and PdCu/P25

Samples	S_{BET} (m^2g^{-1})	Total pore volume (cm^3g^{-1})
P25	37.1	0.27
PdCu/P25	46.8	0.49

Supplementary References:

1. Meng, F. *et al.* Temperature dependent photocatalysis of g-C3N4, TiO2 and ZnO: Differences in photoactive mechanism. *J Colloid Interface Sci* **532**, 321–330 (2018).
2. Balducci, L. *et al.* Direct Oxidation of Benzene to Phenol with Hydrogen Peroxide over a Modified Titanium Silicalite. *Angewandte Chemie International Edition* **42**, 4937–4940 (2003).
3. Yang, J.-H. *et al.* Direct Catalytic Oxidation of Benzene to Phenol over Metal-free Graphene-based Catalyst. *Energy Environ Sci* **6**, 793–798 (2013).
4. Zhu, Y. *et al.* A cocoon silk chemistry strategy to ultrathin N-doped carbon nanosheet with metal single-site catalysts. *Nat Commun* **9**, 3861 (2018).
5. Zhang, T. *et al.* Preassembly Strategy to Fabricate Porous Hollow Carbonitride Spheres Inlaid with Single Cu-N 3 Sites for Selective Oxidation of Benzene to Phenol. *J Am Chem Soc* **140**, 16936–16940 (2018).
6. ElMetwally, A. E., Eshaq, G., Yehia, F. Z., Al-Sabagh, A. M. & Kegnæs, S. Iron Oxychloride as an Efficient Catalyst for Selective Hydroxylation of Benzene to Phenol. *ACS Catal* **8**, 10668–10675 (2018).
7. Pan, Y. *et al.* Regulating the coordination structure of single-atom Fe-NxCy catalytic sites for benzene oxidation. *Nat Commun* **10**, 4290 (2019).
8. Meng, L., Zhu, X. & Hensen, E. J. M. Stable Fe/ZSM-5 Nanosheet Zeolite Catalysts for the Oxidation of Benzene to Phenol. *ACS Catal* **7**, 2709–2719 (2017).
9. Ghosh, S. *et al.* Confined Single Alkali Metal Ion Platform in a Zeolite Pore for Concerted Benzene C-H Activation to Phenol Catalysis. *ACS Catal* **8**, 11979–11986 (2018).
10. Niwa, S. I. *et al.* A one-step conversion of benzene to phenol with a palladium membrane. *Science (1979)* **295**, 105–107 (2002).
11. Tani, M., Sakamoto, T., Mita, S., Sakaguchi, S. & Ishii, Y. Hydroxylation of benzene to phenol under air and carbon monoxide catalyzed by molybdovanadophosphoric acid. *Angewandte Chemie - International Edition* **44**, 2586–2588 (2005).
12. Chen, X., Zhang, J., Fu, X., Antonietti, M. & Wang, X. Fe-g-C3N4-catalyzed oxidation of benzene to phenol using hydrogen peroxide and visible light. *J Am Chem Soc* **131**, 11658–11659 (2009).
13. Devaraji, P., Sathu, N. K. & Gopinath, C. S. Ambient oxidation of benzene to phenol by photocatalysis on Au/Ti0.98V0.02O2: Role of holes. *ACS Catal* **4**, 2844–2853 (2014).
14. Ye, X., Cui, Y., Qiu, X. & Wang, X. Selective oxidation of benzene to phenol by Fe-CN/TS-1 catalysts under visible light irradiation. *Appl Catal B* **152–153**, 383–389 (2014).
15. Wang, D., Wang, M. & Li, Z. Fe-Based Metal-Organic Frameworks for Highly Selective Photocatalytic Benzene Hydroxylation to Phenol. *ACS Catal* **5**, 6852–6857 (2015).
16. Hosseini, S. M. *et al.* Au-Pd@g-C3N4 as an Efficient Photocatalyst for Visible-Light Oxidation of Benzene to Phenol: Experimental and Mechanistic Study. *Journal of Physical Chemistry C* **122**, 27477–27485 (2018).
17. Zhang, Y. & Park, S. J. Stabilizing CuPd bimetallic alloy nanoparticles deposited on holey carbon nitride for selective hydroxylation of benzene to phenol. *J Catal* **379**, 154–163 (2019).

18. Zhang, G., Yi, J., Shim, J., Lee, J. & Choi, W. Photocatalytic hydroxylation of benzene to phenol over titanium oxide entrapped into hydrophobically modified siliceous foam. *Appl Catal B* **102**, 132–139 (2011).
19. Han, J. W., Jung, J., Lee, Y. M., Nam, W. & Fukuzumi, S. Photocatalytic oxidation of benzene to phenol using dioxygen as an oxygen source and water as an electron source in the presence of a cobalt catalyst. *Chem Sci* **8**, 7119–7125 (2017).
20. Chen, P. *et al.* Three-dimension hierarchical heterostructure of CdWO4 microrods decorated with Bi2 WO6 nanoplates for high-selectivity photocatalytic benzene hydroxylation to phenol. *Appl Catal B* **234**, 311–317 (2018).
21. Batista, J., Pintar, A., Gomilšek, J. P., Kodre, A. & Bornette, F. On the structural characteristics of γ-alumina-supported Pd-Cu bimetallic catalysts. *Appl Catal A Gen* **217**, 55–68 (2001).
22. Brun, M., Berthet, A. & Bertolini, J. C. XPS, AES and Auger parameter of Pd and PdO. *J Electron Spectros Relat Phenomena* **104**, 55–60 (1999).
23. Tian, F., Zhang, Y., Zhang, J. & Pan, C. Raman spectroscopy: A new approach to measure the percentage of anatase TiO2 exposed (001) facets. *Journal of Physical Chemistry C* **116**, 7515–7519 (2012).
24. Li, W. S., Shen, Z. X., Li, H. Y., Shen, D. Z. & Fan, X. W. Blue shift of Raman peak from coated TiO2 nanoparticles. *Journal of Raman Spectroscopy* **32**, 862–865 (2001).
25. Mammone, J. F., Sharma, S. K. & Nicol, M. *Raman Study of Rutile (TiO2) at High Pressures*. *Solid State Communications* vol. 34 (1980).
26. Hayashi, T., Nakamura, K., Suzuki, T., Saito, N. & Murakami, Y. OH radical formation by the photocatalytic reduction reactions of H2O2 on the surface of plasmonic excited Au-TiO2 photocatalysts. *Chem Phys Lett* **739**, (2020).
27. Lu, D. *et al.* A facile one-pot synthesis of TiO2-based nanosheets loaded with MnxOy nanoparticles with enhanced visible light-driven photocatalytic performance for removal of Cr(VI) or RhB. *Appl Catal B* **179**, 558–573 (2015).
28. Yu, J., Qi, L. & Jaroniec, M. Hydrogen production by photocatalytic water splitting over Pt/TiO 2 nanosheets with exposed (001) facets. *Journal of Physical Chemistry C* **114**, 13118–13125 (2010).
29. Luan, Y., Jing, L., Wu, J., Xie, M. & Feng, Y. Long-lived photogenerated charge carriers of 001-facet-exposed TiO2 with enhanced thermal stability as an efficient photocatalyst. *Appl Catal B* **147**, 29–34 (2014).
30. Jiao, W. *et al.* Synthesis of mesoporous single crystal rutile TiO2 with improved photocatalytic and photoelectrochemical activities. *Chemical Communications* **49**, 11770–11772 (2013).
