# Supplementary material for: Highly selective oxidation of benzene to phenol with air at room temperature promoted by water
Source: Nat Commun. 2023 Jul 22;14:4431. doi: 10.1038/s41467-023-40160-w (PMC10363151; doi:10.1038/s41467-023-40160-w)
Supplement: Supplementary file 2 — Description of Additional Supplementary Files [file 41467_2023_40160_MOESM2_ESM.docx]

Description: The video shows the dispersion of photocatalyst particles in the water phase and the interface change between benzene and water before and during the reaction.
